# Effect of SNORD113-3/ADAR2 on glycolipid metabolism in glioblastoma via A-to-I editing of PHKA2

**DOI:** 10.1186/s11658-024-00680-9

**Published:** 2025-01-10

**Authors:** Zheng Cui, Xiaobai Liu, Tiange E, Hongda Lin, Di Wang, Yunhui Liu, Xuelei Ruan, Ping Wang, Libo Liu, Yixue Xue

**Affiliations:** 1 Department of Neurosurgery, Shengjing Hospital of China Medical University, Shenyang, 110004 China; 2 Key Laboratory of Neuro-Oncology in Liaoning Province, Shenyang, 110004 China; 3 Liaoning Medical Surgery and Rehabilitation Robot Technology Engineering Research Center, Shenyang, 110004 China; 4 Department of Neurobiology, School of Life Sciences, China Medical University, Shenyang, 110122 China; 5 Department of Neurology, The First Affiliated Hospital, China Medical University, Shenyang, 110001 Liaoning China; 6 Key Laboratory of Neurological Disease Big Data of Liaoning Province, Shenyang, China; 7 Shenyang Clinical Medical Research Center for Difficult and Serious Diseases of the Nervous System, Shenyang, China

**Keywords:** Glioma, Glycolipid metabolism, A-to-I RNA editing, Phosphorylation, ADAR2

## Abstract

**Background:**

Glioblastoma multiforme (GBM) is a highly aggressive brain tumor, characterized by its poor prognosis. Glycolipid metabolism is strongly associated with GBM development and malignant behavior. However, the precise functions of snoRNAs and ADARs in glycolipid metabolism within GBM cells remain elusive. The objective of the present study is to delve into the underlying mechanisms through which snoRNAs and ADARs exert regulatory effects on glycolipid metabolism in GBM cells.

**Methods:**

RNA immunoprecipitation and RNA pull-down experiments were conducted to verify the homodimerization of ADAR2 by SNORD113-3, and Sanger sequencing and Western blot experiments were used to detect the A-to-I RNA editing of PHKA2 mRNA by ADAR2. Furthermore, the phosphorylation of EBF1 was measured by in vitro kinase assay. Finally, in vivo studies using nude mice confirmed that SNORD113-3 and ADAR2 overexpression, along with PHKA2 knockdown, could suppress the formation of subcutaneous xenograft tumors and improve the outcome of tumor-bearing nude mice.

**Results:**

We found that PHKA2 in GBM significantly promoted glycolipid metabolism, while SNORD113-3, ADAR2, and EBF1 significantly inhibited glycolipid metabolism. SNORD113-3 promotes ADAR2 protein expression by promoting ADAR2 homodimer formation. ADAR2 mediates the A-to-I RNA editing of PHKA2 mRNA. Mass spectrometry analysis and in vitro kinase testing revealed that PHKA2 phosphorylates EBF1 on Y256, reducing the stability and expression of EBF1. Furthermore, direct binding of EBF1 to PKM2 and ACLY promoters was observed, suggesting the inhibition of their expression by EBF1. These findings suggest the existence of a SNORD113-3/ADAR2/PHKA2/EBF1 pathway that collectively regulates the metabolism of glycolipid and the growth of GBM cells. Finally, in vivo studies using nude mice confirmed that knockdown of PHKA2, along with overexpression of SNORD113-3 and ADAR2, could obviously suppress GBM subcutaneous xenograft tumor formation and improve the outcome of those tumor-bearing nude mice.

**Conclusions:**

Herein, we clarified the underlying mechanism involving the SNORD113-3/ADAR2/PHKA2/EBF1 pathway in the regulation of GBM cell growth and glycolipid metabolism. Our results provide a framework for the development of innovative therapeutic interventions to improve the prognosis of patients with GBM.

**Supplementary Information:**

The online version contains supplementary material available at 10.1186/s11658-024-00680-9.

## Introduction

Glioblastoma multiforme (GBM) is a malignant brain tumor originating from glial cells. Between 40% and 50% of central nervous system (CNS)-initiated tumors and 80% of CNS malignancies are identified as GBM, and its incidence is continuing to rise global [1]. However, traditional treatment methods such as surgery and chemotherapy generally fail to cure gliomas, with a high recurrence rate and poor prognosis for patients, and the 2-year survival rate is only 15–26% [2, 3]. Metabolic cell reprogramming toward glycolipid metabolism is important in precancerous lesion development in GBM [4]. GBM is significantly connected to abnormal energy metabolism, such as lipid synthesis and aerobic glycolysis, as these provide the raw materials and energy for significant tumor growth [5, 6]. Therefore, antimetabolic therapies for GBM deployed at the molecular level could provide new options for GBM treatment.

The key enzymes associated with glucolipid metabolic reactions can be used as markers for tumor therapy, diagnosis, and prognosis [7]. Pyruvate kinase M2 (PKM2), an enzyme involved in glycolysis, assumes a pivotal position in regulating the rate of glycolysis. In GBM cells, the LINC00689/miR-338-3p/PKM2 axis promotes glycolysis and metastasis [8]. In response to hypoxic conditions, the lncRNA-AC020978 regulates the PKM2/HIF-1 axis in lung cancer cells, thereby enhancing glycolysis and cell growth [9]. In addition, ATP citrate lyase (ACLY) provides a crucial link between cellular lipid metabolism and glucose metabolism. Increased glucose uptake and rapid glycolysis are crucial for energy reprogramming in tumor cells, leading to elevated ACLY levels and promoting fatty acid synthesis. Furthermore, the IKKβ–USP30–ACLY axis could regulate carcinogenesis and adipogenesis significantly [10]. ACLY is upregulated in glioma cells and regulates their adherence and invasion [11]. Therefore, elucidating the molecular mechanism of regulation of the two key glucose and lipid metabolism enzymes, ACLY and PKM2, in GBM may be crucial for further exploring reliable biomarkers.

SnoRNAs represent a category of small noncoding RNAs primarily situated within the nucleus, harboring conserved structural motifs. Ribosomal RNA (rRNA) precursors are processed, spliced, and modified after transcription by these snoRNAs [12]. Recently, it has been demonstrated that snoRNAs participate in glucose and lipid metabolism, in addition to playing a role in the development of GBM. For example, SNORD12B can promote glycolipid metabolism and proliferation of GBM cells [13], whereas SNORD76 can inhibit the proliferation of GBM cells [14].

It is an important factor in tumor heterogeneity that A-to-I RNA editing occurs in mammalian CNS [15]. RNA editing from A-to-I is catalyzed by ADAR enzymes (such as ADAR1–3) [16]. It has been demonstrated that snoRNA serves a crucial function in the process of A-to-I editing. For example, snord115 increases the activity of pre mRNA of HTR2C by A-to-I editing [17]. Cell migration and invasion are modulated by several ADAR2 substrates during partial GBM pathogenesis. For instance, the enhanced RNA editing of miR-589-3p from A to I catalyzed by ADAR2 can effectively suppress the migratory and invasive capabilities of GBM cells [18]. Moreover, a recent study found that A-to-I editing is involved in aerobic glycolysis [19].

Phosphorylase kinase regulatory subunit alpha 2 (PHKA2) executes the function of phosphorylase kinase. Hepatocyte glucose metabolism is regulated by PHKA2, and high expression of PHKA2 would lead to excessive hepatic glycogen storage [20]. In addition, recent studies have demonstrated that EBF transcription factor 1 (EBF1) regulates the development and progression of tumors. Additionally, EBF1 promotes breast cancer progression through the HIF1α pathway [21], and inhibition of thyroid cancer progression by noncoding RNA LINC00261 can be achieved through the regulation of EBF1 [22].

Recent evidence suggests that glycolipid metabolism-related transcription factors are potential new cancer targets, and they have been evaluated in preclinical trials [7]. For example, the phosphorylation of transcription factor JMJD55 can regulate aerobic glycolysis of GBM cells through PKM2 [23]. Therefore, molecular regulatory pathways composed of snoRNAs, ADARs, and transcription factors may participate in GBM occurrence and development by regulating key enzymes of glucose and lipid metabolism. Knowledge of the molecular mechanisms behind the regulatory effects of such factors on GBM cell growth and glycolipid metabolism can provide valuable insights into the processes underlying glycolipid metabolism and GBM development and facilitate identification of novel molecular targets for GBM treatment.

## Materials and methods

### Clinical specimens

We obtained normal brain and glioma specimens from China Medical University’s Affiliated Hospital for Neurosurgery. This was approved by the Ethics Committee of China Medical University’s affiliated Shengjing Hospital, and all patients or their families signed the informed consent form (approval no. 2023PS957K).

### Cell cultures

Glioma cell lines, specifically U87, A172, U251, and U373, were furnished by the Biological Sciences Cell Resource Center situated in Shanghai, China. Additionally, normal human astrocytes (NHA) were supplied by ScienCell Research Laboratories, located in California, USA. All cells were strictly cultured according to American Type Culture Collection guidelines (https://www.atcc.org/). All cells were identified using short tandem repeats and cell line authentication.

### Quantitative real-time PCR (qRT–PCR)

The specific experimental method of qRT-PCR is as described in the previous article [6]. The primers utilized in the research are comprehensively outlined in Supplementary Table S1. For further clarification, kindly refer to the Supporting Information section pertaining to Materials and Methods.

### Short hairpin RNA (shRNA) synthesis

The following plasmids were meticulously synthesized by GeneChem (Shanghai, China): SNORD113-3( +), SNORD113-3(–), and their negative controls (NCs) SNORD113-3( +)NC and SNORD113-3(–)NC; ADAR2(–), ADAR2( +), and their NCs ADAR2(–)NC and ADAR2( +)NC; ADAR1(–) and its NC ADAR1(–)NC; PHKA2( +), PHKA2(–), and their NCs PHKA2( +)NC and PHKA2(–)NC; EBF1( +), EBF1(–), and their NCs EBF1( +)NC and EBF1(–)NC. Successful transfection was ascertained through qRT-PCR or western blot analysis (Supplementary Figs. S1–6).

The following plasmids were synthesized by GenePharma (Shanghai, China): EBF1-WT, EBF1-Y256A, EBF1-Y256E, and tRSA-SNORD113-3. Prokaryotic expression plasmids fused with GST, HA, or FLAG tag were also produced by GenePharma: FLAG-ADAR2, FLAG-PHKA2, HA-ADAR2, GST-EBF1(-WT), and GST-EBF1-Y256A.

### Western blot

Western blotting was conducted in accordance with the previously published article [6]. For a comprehensive understanding of the experimental procedures and the antibodies employed, kindly refer to the Supporting Information section pertaining to Materials and Methods.

### Extracellular acidification rate (ECAR) measurement

The ECAR analysis was conducted in accordance with the previously published article [6]. For further clarification, kindly refer to the Supporting Information section pertaining to Materials and Methods.

### Metabolic parameters of glucose utilization and lactate production

The metabolic parameters of glucose uptake and lactate production were evaluated in accordance with the previously published article [6]. For further clarification, kindly refer to the Supporting Information section pertaining to Materials and Methods.

### Dual-luciferase reporter assay

The assay in this research followed procedures previously reported [6]. For further clarification, kindly refer to the Supporting Information section pertaining to Materials and Methods.

### Sequencing of the PHKA2 3′-UTR in glioma cells

To identify potential A-to-I RNA editing occurrences within the 3′-UTR region of PHKA2 in GBM cells, cDNA was synthesized from glioma RNA employing the PrimeScript RTase enzyme sourced from Takara Bio Inc., located in Kusatsu, Japan. Specifically designed primers, detailed in Supplementary Table S1, were utilized to specifically target the 3′-UTR region of PHKA2. These primers were then employed in the amplification of a glioma cDNA fragment, and the resulting PCR product underwent Sanger sequencing for further analysis.

### RNA immunoprecipitation (RIP) assay

The RIP assay in this research followed procedures previously reported [6]. For further clarification, kindly refer to the Supporting Information section pertaining to Materials and Methods.

### Cell proliferation assay

The cell proliferation assay in this research followed procedures previously reported [6]. For further clarification, kindly refer to the Supporting Information section pertaining to Materials and Methods.

### Lipid droplet staining and quantification

Lipid droplet staining and quantification was conducted in accordance with the previously published article [13]. For further clarification, kindly refer to the Supporting Information section pertaining to Materials and Methods.

### Colorimetric triglyceride and cholesterol measurement assays

The determination of triglycerides was conducted by utilizing an available quantitative triglyceride assay kit (Nanjing Jiancheng Biological Engineering Ltd, Nanjing, China). In summary, upon the addition of lipase, triglycerides undergo a conversion process, resulting in the formation of glycerol and free fatty acids. Subsequently, glycerol reacts with oxidase, leading to the generation of hydrogen peroxide. This hydrogen peroxide then interacts with the commercial probe, giving rise to a colored product that can be accurately detected by the optical density of 510 nm. Quantification of total cholesterol was conducted using a commercially available assay kit from Nanjing Jiancheng Biological Engineering Ltd., adhering to a similar methodology. Absorbencies were measured using a SpectraMaxM5 microplate reader from Molecular Devices, USA. To standardize the findings, the final triglyceride or cholesterol levels were calibrated using the total protein quantity measured via a BCA protein assay.

### Gene expression profiles

Stable SNORD113-3 or ADAR2 overexpression cells and their glioma counterparts were analyzed for genome-wide expression profiling. KangChen Biotech in Shanghai, China performed whole-genome profiling using Agilent microarrays. The RNA samples were amplified and then transcribed into cDNA, followed by hybridization to microarrays. Subsequently, the microarrays were washed and scanned. Genes exhibiting a twofold expression change and a *P*-value less than 0.05 were deemed to be differentially expressed.

### Fluorescence in situ hybridization (FISH) assay

The FISH assay was conducted in accordance with the previously published article [6]. For further clarification, kindly refer to the Supporting Information section pertaining to Materials and Methods.

### Immunofluorescence (IF) assay

The IF assay in this research followed procedures previously reported [24]. For further clarification, kindly refer to the Supporting Information section pertaining to Materials and Methods.

### RNA pull-down assays

The RNA pull-down assay in this research followed procedures previously reported [24]. For further clarification, kindly refer to the Supporting Information section pertaining to Materials and Methods.

### Chromatin immunoprecipitation (ChIP) assay

The ChIP assay in this research followed procedures previously reported [24]. For further clarification, kindly refer to the Supporting Information section pertaining to Materials and Methods. Immunoprecipitated DNA was amplified using primers in Supplementary Table S3.

### Co-immunoprecipitation assay

The co-IP assay in this research followed procedures previously reported [24]. For further clarification, kindly refer to the Supporting Information section pertaining to Materials and Methods.

### GST pull-down assays

The following prokaryotic plasmids were constructed, expressing a protein of interest that has been fused with either a FLAG or glutathione-*S*-transferase (GST) tag. These plasmids include FLAG-PHKA2, GST-EBF1-WT, and GST-EBF1-Y256A. To conduct GST pull-down experiments, purified FLAG-PHKA2 protein was incubated overnight at 4 °C with purified GST-EBF1 proteins that were bound to resin. Subsequently, the eluted protein complexes were subjected to analysis using SDS-PAGE and western blot, as previously described.

### In vitro protein kinase assays

The in vitro protein kinase assays was conducted in accordance with the previously published article [24]. For further clarification, kindly refer to the Supporting Information section pertaining to Materials and Methods.

### Mass spectrometry analysis

The proteins were separated via SDS-PAGE gel electrophoresis, with subsequent excision of the target protein bands from the gel. Following elution, reduction, and alkylation procedures, the proteins underwent overnight digestion at a controlled temperature of 37 °C. The resulting peptides were then collected, desalted, and subjected to analysis using a state-of-the-art timstoFpro mass spectrometer sourced from Bruker in Bremen, Germany. The sequences and sites of interest were accurately identified through the utilization of the BLAST tool available on the NCBI platform.

### RNA stability evaluation

The RNA stability evaluation assays was conducted in accordance with the previously published article [6]. For further clarification, kindly refer to the Supporting Information section pertaining to Materials and Methods.

### Cycloheximide chase assay

The half-lives of EBF1 and ADAR2 were accurately measured through the utilization of the protein synthesis inhibitor, cycloheximide. Specifically, U251 cells transfected with various EBF1 constructs, namely EBF1-WT, EBF1-Y256A, and EBF1-Y256E, were treated with a concentration of 100 mg/mL of cycloheximide (supplied by Beijing NobleRyder Science and Technology Company). Subsequently, these cells were collected at designated time points, namely 0, 2, 4, 8, and 10 h. Subsequently, ten total protein extracts were meticulously prepared and underwent immunoblotting, adhering strictly to the previously outlined protocol.

### Tumor xenografts in nude mice

A xenograft models were established by using the constructed stably transfected glioma cells (U251 and U373) in nude mice. For further clarification, kindly refer to the Supporting Information section pertaining to Materials and Methods.

### Statistical analysis

All data are presented as mean ± standard deviation (SD). Statistical analyses were conducted utilizing GraphPad Prism version 8.4 (GraphPad, California, USA). For comparisons involving two groups, *T*-tests were employed. Conversely, for comparing multiple groups, one-way or two-way ANOVA was used. Statistical significance was determined with a *P*-value threshold of < 0.05.

## Results

### SNORD113-3 is downregulated in GBM tissues and cells and suppresses glycolipid metabolism and GBM cell proliferation

**Fig. 1:**
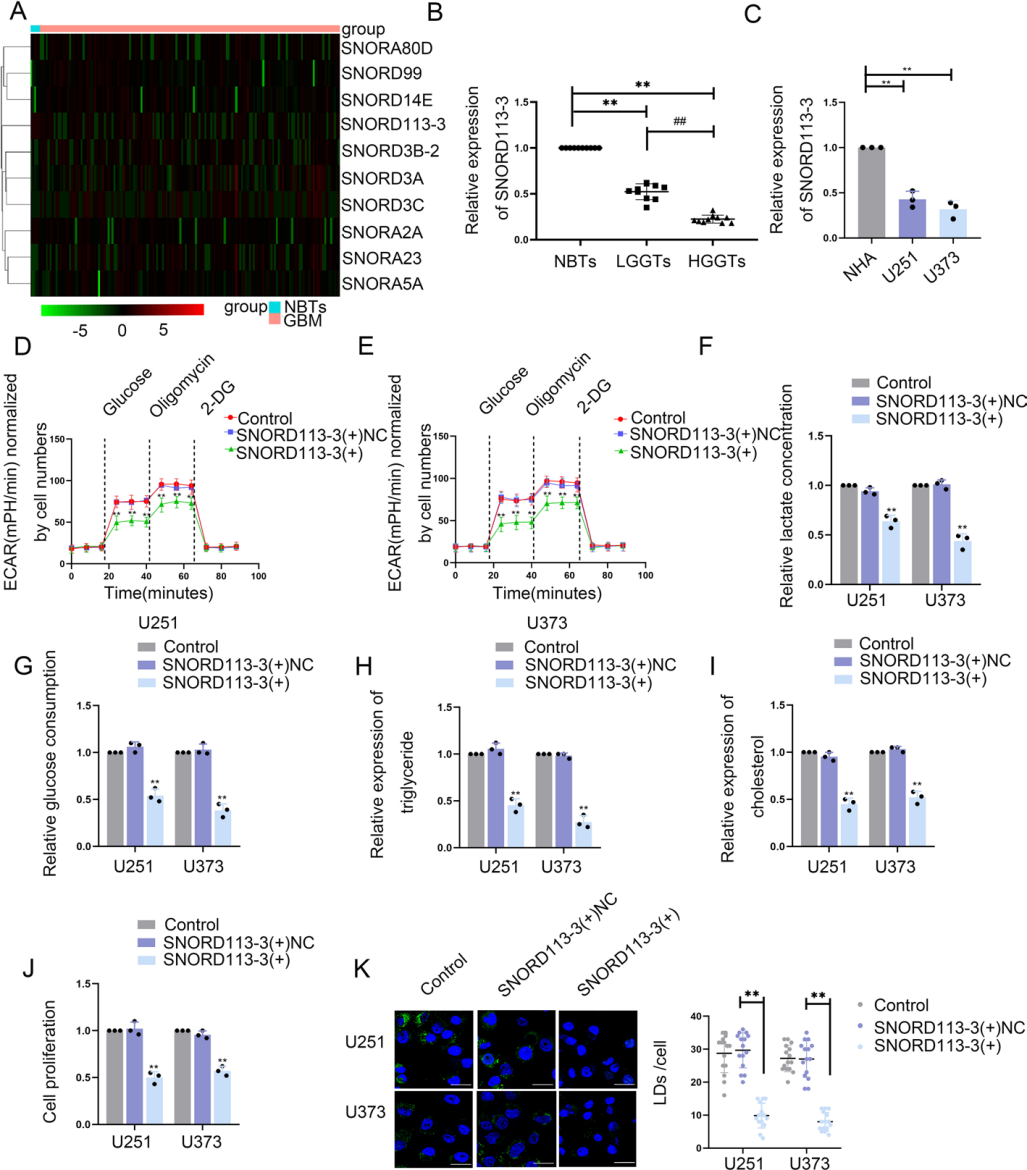
Screening for SNORD113-3, the expression of SNORD113-3, and its effects on glycolipid metabolism in GBM. **A** Heatmap with hierarchical cluster analysis of differentially expressed snoRNAs between non-tumor brain tissues (NBTs) and glioblastoma (GBM) tissues. *P* < 0.05, |log2FC|> 1. **B** SNORD113-3 expression was downregulated in tissues (NBTs [*n* = 10], LGGTs [*n* = 10], and HGGTs [*n* = 10]) via qRT-PCR. ^**^P < 0.01 versus NBTs group; ^##^*P* < 0.01 versus LGGTs group. **C** SNORD113-3 expression level was analyzed in NHA cell, U251, and U373 cells via qRT-PCR. Data presented as mean ± SD (*n* = 3, each group), ^**^P < 0.01 versus the NHA group. **D**, **E** The extracellular acidification rate (ECAR) was measured to demonstrate the effects of SNORD113-3 on glycolysis in U251 and U373 cells, and the glycolysis was calculated. Data presented as mean ± SD (*n* = 3, each group), ^**^P < 0.01 versus SNORD113-3( +)NC group. **F**, **G** Lactate production and glucose uptake were measured in U251 and U373 cells after SNORD113-3 overexpression. Data presented as mean ± SD (*n* = 3, each group), ^**^P < 0.01 versus SNORD113-3( +)NC group. **H**, **I** Intracellular triglyceride and cholesterol expression levels were measured after SNORD113-3 overexpression. Data presented as mean ± SD (*n* = 3, each group), ^**^P < 0.01 versus SNORD113-3( +)NC group. **J** The effect of SNORD113-3 on proliferation was analyzed via CCK-8 assay. Data presented as mean ± SD (*n* = 3, each group), ^**^P < 0.01 versus SNORD113-3( +)NC group. **K** Representative confocal fluorescence imaging of lipid droplets (LDs) stained by BODIPY 493/503 (green) in U251 and U373 cells. The nucleus (blue) was stained by DAPI. Scale bars = 10 µm. Data presented as mean ± SD (*n* = 15, each group). ^**^P < 0.01 versus SNORD113-3( +)NC group. One-way ANOVA was used for statistical analysis

The TCGA database (https//:portal.gdc.cancer.gov/) was utilized to discern differentially expressed snoRNAs in comparison between GBM and normal brain tissues (NBTs) (Fig. 1A, Supplementary Table S2). To investigate the impact of these snoRNAs on glucose uptake and lactate production, they were knocked down in U373 and U251 cells. Among them, SNORD113-3 was chosen for further examination (Fig. S1A, B). qRT-PCR revealed that, compared with NHA, GBM cells, such as U87, U251, U373, and A172, presented decreased SNORD113-3 expression. U251 and U373 cell lines were selected for subsequent experiments (Supplementary Fig. S1C). The qRT-PCR analysis has demonstrated a significant reduction in the expression level of SNORD113-3 in GBM tissues, in comparison with its expression in NBT tissues. Additionally, a consistent decrease in SNORD113-3 expression was observed in correlation with the escalating pathological grade of glioma (Fig. 1B). Compared with NHAs, the expression of SNORD113-3 was notably downregulated in both U251 and U373 cells (Fig. 1C). Furthermore, confocal microscopy provided evidence of the nuclear localization of SNORD113-3 in U251 and U373 cells (Supplementary Fig. S1D).

An investigation was conducted into the impact of SNORD113-3 on glycolipid metabolism and the proliferation of GBM cells. First, U251 and U373 cell lines with stable SNORD113-3 overexpression were constructed, and the group with the highest transfection efficiency was selected for further study (Supplementary Fig. S1E). Western blot was then conducted to measure the PKM2 and ACLY levels at the translational level in SNORD113-3-overexpression cells. PKM2 and ACLY expression was significantly reduced in SNORD113-3-overexpression cells (Supplementary Fig. S1F). We then measured the extracellular acidification rate, lactate, glucose, triglyceride, and cholesterol levels, and cell proliferation capacity of SNORD113-3-overexpression cells. In SNORD113-3-overexpression U251 and U373 cells, the aerobic glycolysis, lactate production, glucose utilization rate, lipid metabolism, and proliferation capacity were significantly reduced (Fig. 1D–J). Furthermore, fluorescence staining using BODIPY493/503 showed significantly reduced lipid droplets in U251 and U373 cells after SNORD113-3 overexpression (Fig. 1K).

### ADAR2 is downregulated in GBM tissues and cells and suppresses glycolipid metabolism and GBM cell proliferation

**Fig. 2:**
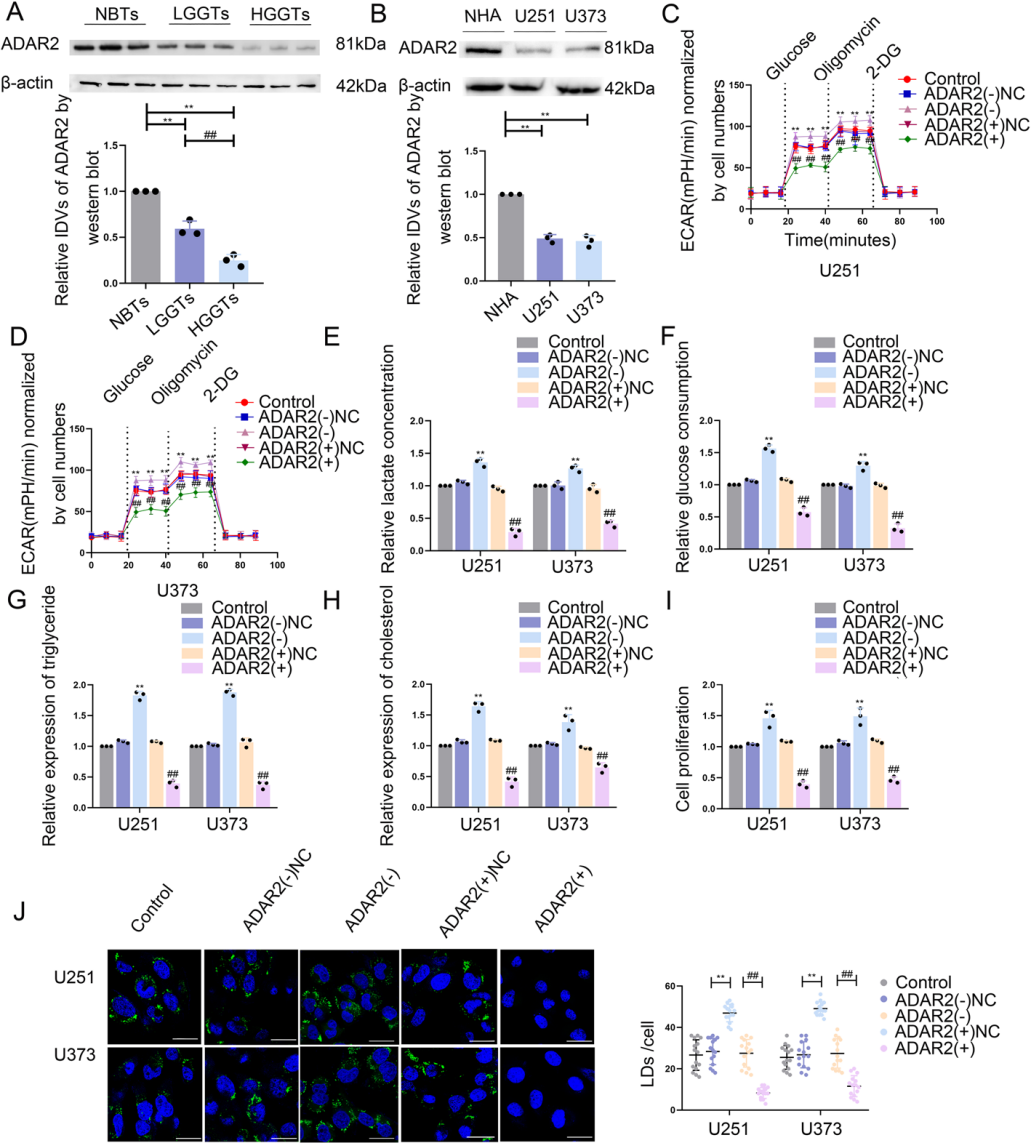
The expression of ADAR2 and its effects on glycolipid metabolism and proliferation in GBM. **A** ADAR2 protein levels were analyzed in NBTs, LGGTs, and HGGTs by western blot. Data presented as mean ± SD (*n* = 3, each group), ^**^P < 0.01 versus NBTs group; ^##^*P* < 0.01 versus LGGTs group. **B** ADAR2 protein levels in NHA, U251, and U373 cells were detected via western blot. Data presented as mean ± SD (*n* = 3, each group), ^**^P < 0.01 versus the NHA group. **C**, **D** The extracellular acidification rate (ECAR) was measured to demonstrate the effects of ADAR2 on glycolysis in U251 and U373 cells, and the glycolysis was calculated. Data presented as mean ± SD (*n* = 3, each group), ^**^P < 0.01 versus ADAR2(–)NC group; ^##^*P* < 0.01 versus ADAR2( +)NC group. **E**, **F** Lactate production and glucose uptake were measured in U251 and U373 cells after ADAR2 knockdown or overexpression. Data presented as mean ± SD (*n* = 3, each group), ^**^P < 0.01 versus ADAR2(–)NC group; ^##^*P* < 0.01 versus ADAR2( +)NC group. **G**, **H** Intracellular triglyceride and cholesterol expression levels were measured to evaluate the effect of ADAR2 on lipogenesis. Data presented as mean ± SD (*n* = 3, each group), ^**^P < 0.01 versus ADAR2(–)NC group; ^##^*P* < 0.01 versus ADAR2( +)NC group. **I** The effect of ADAR2 on proliferation was analyzed via CCK-8 assay. Data presented as mean ± SD (*n* = 3, each group), ^**^P < 0.01 versus ADAR2(–)NC group; ^##^*P* < 0.01 versus ADAR2( +)NC group. **J** Representative confocal fluorescence imaging of LDs stained by BODIPY 493/503 (green) in U251 and U373 cells after ADAR2 knockdown or overexpression. The nucleus (blue) was stained by DAPI. Scale bars = 10 µm. Data presented as mean ± SD (*n* = 15, each group). ^**^P < 0.01 versus ADAR2(–)NC group; ^##^*P* < 0.01 versus ADAR2( +)NC group. Statistical analysis was performed using the one-way ANOVA method

SNORD113-3 overexpression in U251 and U373 cells was associated with the upregulation of several genes as revealed by microarray analysis (Supplementary Fig. S2A). The expression level of mRNA for the RNA editing enzyme ADAR2 was increased significantly in U251 and U373 cells that overexpressed SNORD113-3, exhibiting a robust association with glucose uptake and lactate production. Consequently, ADAR2 was selected for further investigation (Supplementary Fig. S2B–E). Additionally, significant nuclear localization of ADAR2 in GBM cells was revealed by IF staining (Supplementary Fig. S2F). Western blots revealed that ADAR2 protein was more deficient in GBM tissues than in NBTs, and the decrease was more pronounced in higher pathological grades (Fig. 2A). Compared with NHAs, ADAR2 expression was downregulated significantly in both U251 and U373 cells (Fig. 2B). To further investigate the regulatory impact of ADAR2 on glycolipid metabolism in GBM cells, stable knockdown or overexpression of ADAR2 was successfully achieved in the U251 and U373 cell lines (transfection efficiency is shown in Supplementary Fig. S2G, H). ADAR2 overexpression resulted in dramatically reduced PKM2 and ACLY expression at the protein level (Supplementary Fig. S2I) and led to a notable reduction in the aerobic glycolysis, lactate production, glucose utilization, lipogenesis, and proliferation of GBM cells (Fig. 2C–J). Conversely, ADAR2 knockdown increased PKM2 and ACLY expression at the protein level significantly (Supplementary Fig. S2G) and promoted GBM cell growth and glycolipid metabolism (Fig. 2C–J).

### SNORD113-3 promotes ADAR2 dimerization, glycolipid metabolism, and GBM cell proliferation

**Fig. 3:**
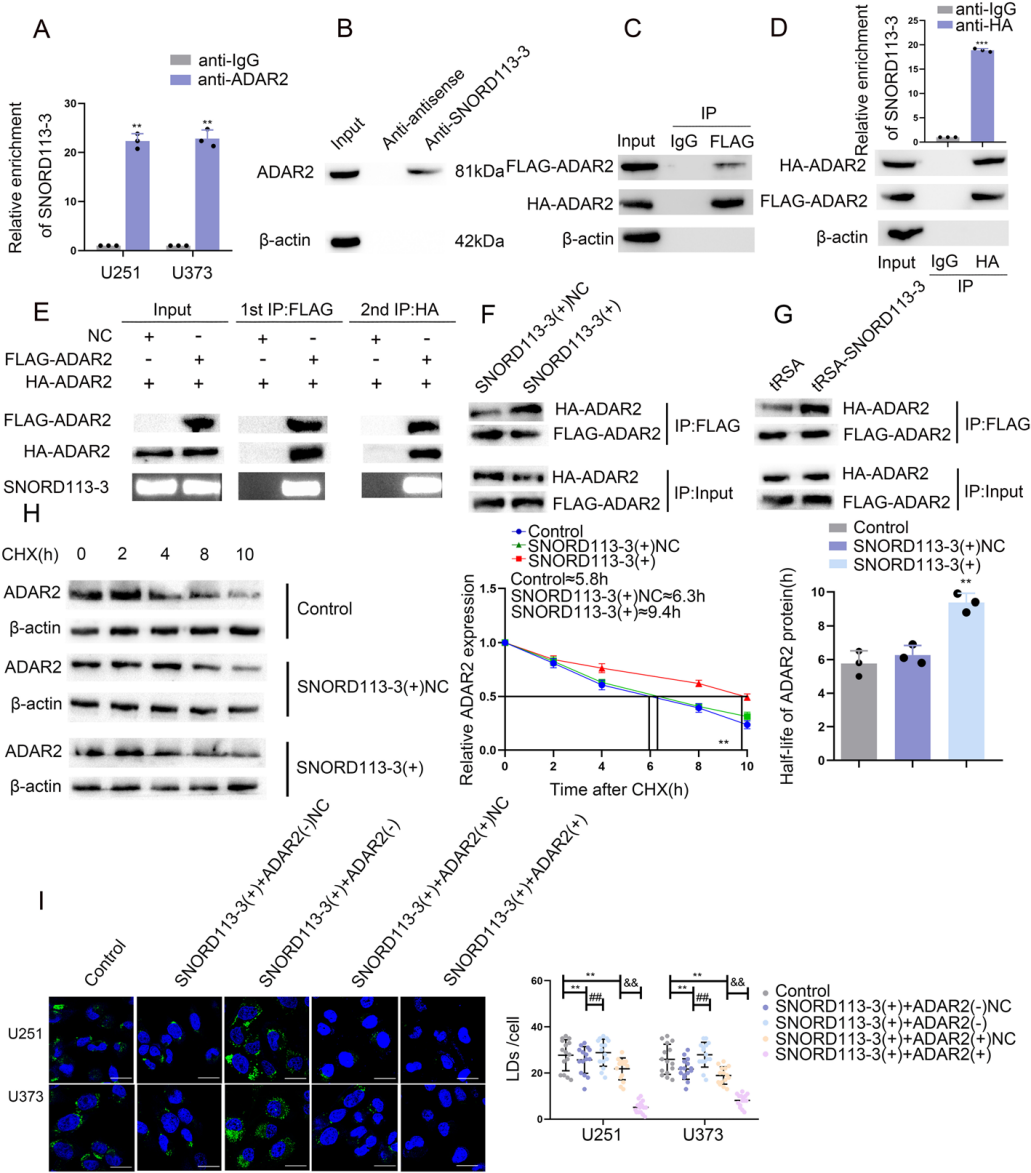
SNORD113-3 facilitated glycolipid metabolism of GBM cells by promoting ADAR2 dimerization. **A** An enrichment of SNORD113-3 in ADAR2 immunoprecipitated samples via RNA immunoprecipitation (RIP) assay. Data presented as mean ± SD (*n* = 3, each group), ^**^P < 0.01 versus the anti-IgG group. **B** RNA pull-down assay followed by western blot showed the specific associations of ADAR2 with biotinylated-SNORD113-3 or antisense RNA. **C** Immunoprecipitation of FLAG-ADAR2 with HA-ADAR2 was performed using 293 T cells. **D** FLAG-ADAR2 and SNORD113-3 were coprecipitated with HA-ADAR2 in 293 T cells. **E** FLAG-ADAR2, HA-ADAR2, and SNORD113-3 were coprecipitated with anti-FLAG antibody, eluted with FLAG peptide, and then coprecipitated with the anti-HA antibody (*n* = 3, each group). **F**, **G** SNORD113-3 promote the binding of FLAG-ADAR2 to HA-ADAR2 both in vivo and in vitro (*n* = 3, each group). **H** The effects of ADAR2 stability were detected by cycloheximide (CHX) chase assays. Data presented as mean ± SD (*n* = 3, each group). ^**^P < 0.01 versus SNORD113-3( +)NC group. **I** Representative confocal fluorescence imaging of LDs stained by BODIPY 493/503 (green) in U251 and U373 cells. The nucleus (blue) was stained by DAPI. Scale bars = 10 µm. Data presented as mean ± SD (*n* = 15, each group). ^**^P < 0.01 versus control group; ^##^*P* < 0.01 versus SNORD113-3( +) + ADAR2(–)NC group; ^*&&*^*P* < 0.01 versus SNORD113-3( +) + ADAR2( +)NC group. Statistical analysis was performed using the one-way ANOVA method

We conducted an investigation to delve into the association between SNORD113-3 and ADAR2. The bioinformatics database, starBasev2.0 (https://starbase.sysu.edu.cn/), indicates the existence of a potential binding interaction between ADAR2 and SNORD113-3. RIP showed SNORD113-3 enrichment in the ADAR2-immunoprecipitated samples compared with those immunoprecipitated using IgG (Fig. 3A). RNA pull-down experiments detected the binding of ADAR2 to SNORD113-3 in U251 cells (Fig. 3B). The data suggest that SNORD113-3 binds directly to ADAR2. While SNORD113-3 is indeed classified as a C/D box snoRNA, whose typical function involves 2′-O methylation within the nucleolus, our analysis utilizing NmSEER 2.0 prediction revealed no identifiable 2′-O methylation sites at either SNORD113-3 or ADAR2 binding locations (http://www.rnanut.net/nmseer-v2/). This suggests that SNORD113-3 may affect the nucleus through other molecular mechanisms independent of the rRNA modification induced by snoRNA.

The dsRBDs of ADAR2 can directly bind to and edit dsRNAs; however, no editing sites for SNORD113-3 were identified in the RNA editing databases DARNED (http://darned.ucc.ie/about/) and REDIportal (http://srv00.recas.ba.infn.it/atlas/), indicating that SNORD113-3 is not a target gene for ADAR2 editing. As ADAR2 RNA editing depends on homodimerization, we wondered whether SNORD113-3 affected ADAR2 expression via ADAR2 homodimerization. The fundamental assumption underlying this conjecture is the capacity of SNORD113-3 to establish an association with an ADAR2 dimer. We first found that Flag-ADAR2 was coprecipitated with HA-ADAR2 in 293 T cells by co-IP assays (Fig. 3C). The simultaneous overexpression of FLAG-ADAR2, HA-ADAR2, and SNORD113-3 in 293 T cells followed by HA-ADAR2 immunoprecipitation assay showed significant enrichment of both FLAG-ADAR2 and SNORD113-3 (Fig. 3D). Furthermore, a rigorous two-step RIP analysis revealed that, during the first phase, the application of anti-FLAG antibody effectively coprecipitated HA-ADAR2 and SNORD113-3. Subsequently, the use of anti-HA antibody resulted in the concurrent precipitation of HA-ADAR2, FLAG-ADAR2, and SNORD113-3 (Fig. 3E). These experiments demonstrate that the dimer of SNORD113-3 and ADAR2 form a functional structure.

The authors further explored whether SNORD113-3 affects the dimerization of ADAR2. The results indicated that SNORD113-3 overexpression resulted in elevated relative expression of HA-ADAR 2 coupled to FLAG-ADAR 2 in either U251 cells or 293 T cells (Fig. 3F, G), indicating that SNORD113-3 promotes ADAR2 dimerization. Furthermore, upon examination of the results obtained from the cycloheximide chase assay, it was observed that the half-life of ADAR2 was significantly extended following the overexpression of SNORD113-3 (Fig. 3H), further confirming that SNORD113-3 is able to increase ADAR2 stability by promoting the formation of ADAR2 dimers and subsequently increasing ADAR2 protein expression.

The potential involvement of ADAR2 in the effect of SNORD113-3 expression on GBM cell glycolipid metabolism and proliferation was also investigated. After transfection of ADAR2(–), ADAR2( +), or the NC plasmids into stable SNORD113-3-overexpression cells, the ADAR2-overexpressed cells showed significantly reduced PKM2 and ACLY expression, glycolipid metabolism, and GBM cell proliferation (Supplementary Figs. 3I and S3A–H). However, the knockdown of ADAR2 reversed the suppressive effect exerted by SNORD113-3 on the expression of PKM2 and ACLY, resulting in the augmentation of glycolipid metabolism and the enhancement of GBM cell proliferation (Supplementary Figs. 3I and S3A–H).

### PHKA2 upregulation in GBM tissues and cells and PHKA2 knockdown inhibits glycolipid metabolism and GBM cell proliferation

Microarray analysis revealed several significantly downregulated genes in ADAR2-overexpressed U251 and U373 cells (Supplementary Fig. S4A). The expression level of PHKA2 was decreased notably in U251 and U373 cells, and its downregulation was closely associated with glucose uptake and lactate production (Supplementary Fig. S4B–E). The RNA editing databases DARNED (http://darned.ucc.ie/about/) and REDIportal (http://srv00.recas.ba.infn.it/atlas/) have demonstrated that the process of A-to-I RNA editing can potentially take place within the 3′-UTR region of PHKA2 mRNA. Therefore, PHKA2 was selected for further study.

**Fig. 4:**
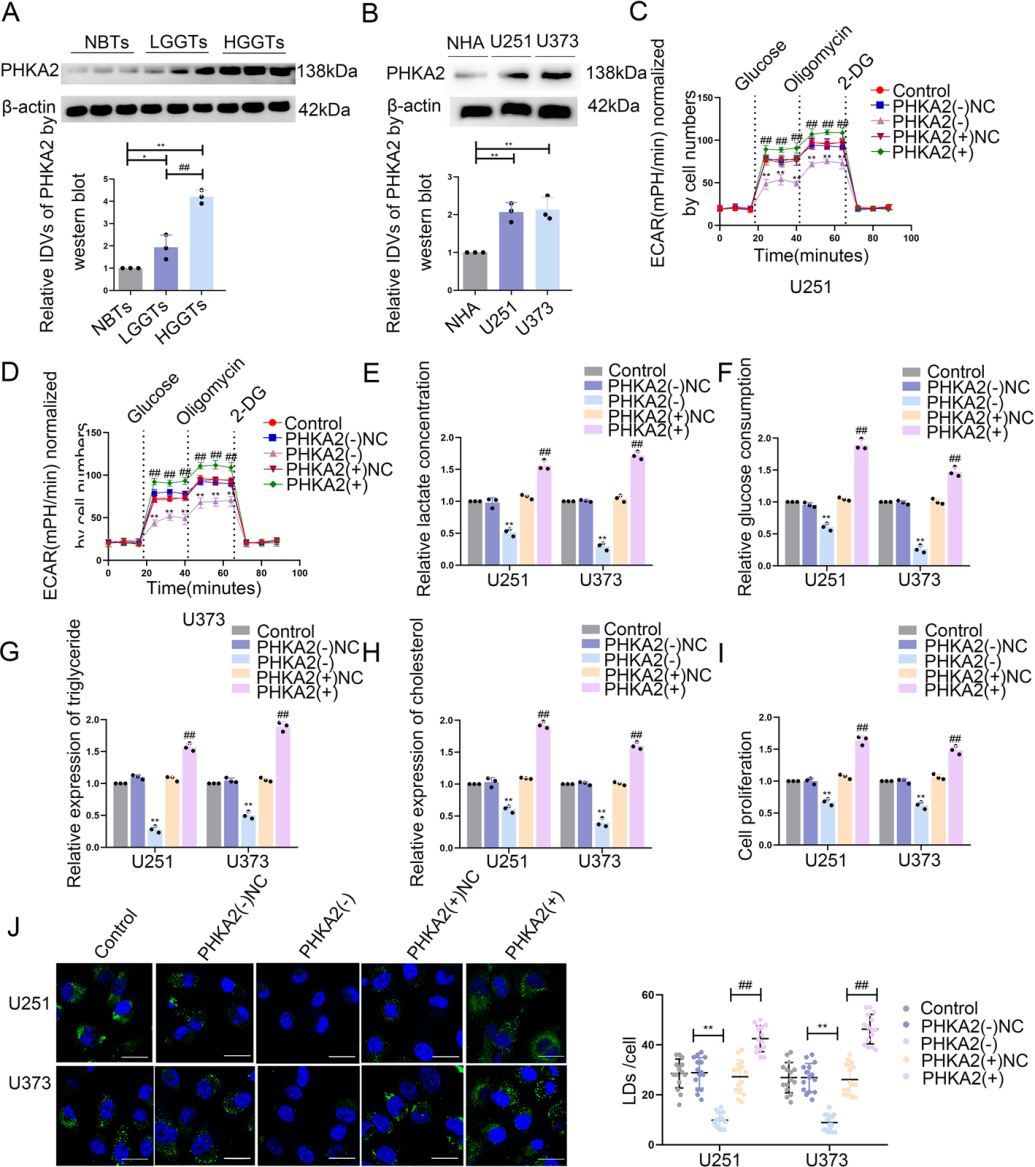
The expression of PHKA2 and its effects on glycolipid metabolism in GBM. **A** PHKA2 protein levels were analyzed in NBTs, LGGTs, and HGGTs by western blot. Data presented as mean ± SD (*n* = 3, each group), ^*^P < 0.05 versus NBTs group; ^**^P < 0.01 versus NBTs group; ^##^*P* < 0.01 versus LGGTs group. **B** PHKA2 protein levels in NHA, U251, and U373 cells were detected via western blot. Data presented as mean ± SD (*n* = 3, each group), ^**^P < 0.01 versus the NHA group. **C**, **D** The extracellular acidification rate (ECAR) was measured to demonstrate the effects of PHKA2 on glycolysis in U251 and U373 cells, and the glycolysis was calculated. Data presented as mean ± SD (*n* = 3, each group), ^**^P < 0.01 versus PHKA2(–)NC group; ^##^*P* < 0.01 versus PHKA2( +)NC group. **E**, **F** Lactate production and glucose uptake were measured in U251 and U373 cells after PHKA2 knockdown or overexpression. Data presented as mean ± SD (*n* = 3, each group), ^**^P < 0.01 versus PHKA2(–)NC group; ^##^*P* < 0.01 versus PHKA2( +)NC group. **G**, **H** Intracellular triglyceride and cholesterol expression levels were measured to evaluate the effect of PHKA2 on lipogenesis. Data presented as mean ± SD (*n* = 3, each group), ^**^P < 0.01 versus PHKA2(–)NC group; ^##^*P* < 0.01 versus PHKA2( +)NC group. **I** The effect of PHKA2 on proliferation was analyzed via CCK-8 assay. ^**^P < 0.01 versus PHKA2(–)NC group; ^##^*P* < 0.01 versus PHKA2( +)NC group. Data presented as mean ± SD (*n* = 3, each group). **J** Representative confocal fluorescence imaging of lipid droplets (LDs) stained by BODIPY 493/503 (green) in U251 and U373 cells. The nucleus (blue) was stained by DAPI. Scale bars = 10 µm. Data presented as mean ± SD (*n* = 15, each group). ^**^P < 0.01 versus PHKA2(–)NC group; ^##^*P* < 0.01 versus PHKA2( +)NC group. Statistical analysis was performed using the one-way ANOVA method

IF staining showed the PHKA2 cytoplasm localization in GBM cells (Supplementary Fig. S4F). Compared with NBTs, glioma tissues showed significantly higher PHKA2 expression, positively correlating with pathological grades (Fig. 4A). Consistent with the pattern observed in GBM tissues, GBM cells, including U251 and U373, demonstrated notably elevated PHKA2 expression levels (Fig. 4B). To elucidate the role of PHKA2 in glycolipid metabolism and GBM cell proliferation, we have established U251 and U373 cell lines in which PHKA2 has been knocked down or overexpressed. The levels of PHKA2 mRNA and protein expression within the cell lines are presented in Supplementary Fig. S4G, H. PKM2 and ACLY expression was reduced significantly in PHKA2-knockdown cells and suppressed glycolipid metabolism and GBM cell growth dramatically (Supplementary Fig. S4I; Fig. 4C–J). Conversely, PHKA2 overexpression enhanced PKM2 and ACLY expression, glycolipid metabolism, and GBM cell proliferation (Supplementary Fig. S4I; Fig. 4C–J).

### ADAR2 regulates PHKA2 expression by mediating A-to-I RNA editing of PHKA2 mRNA

**Fig. 5:**
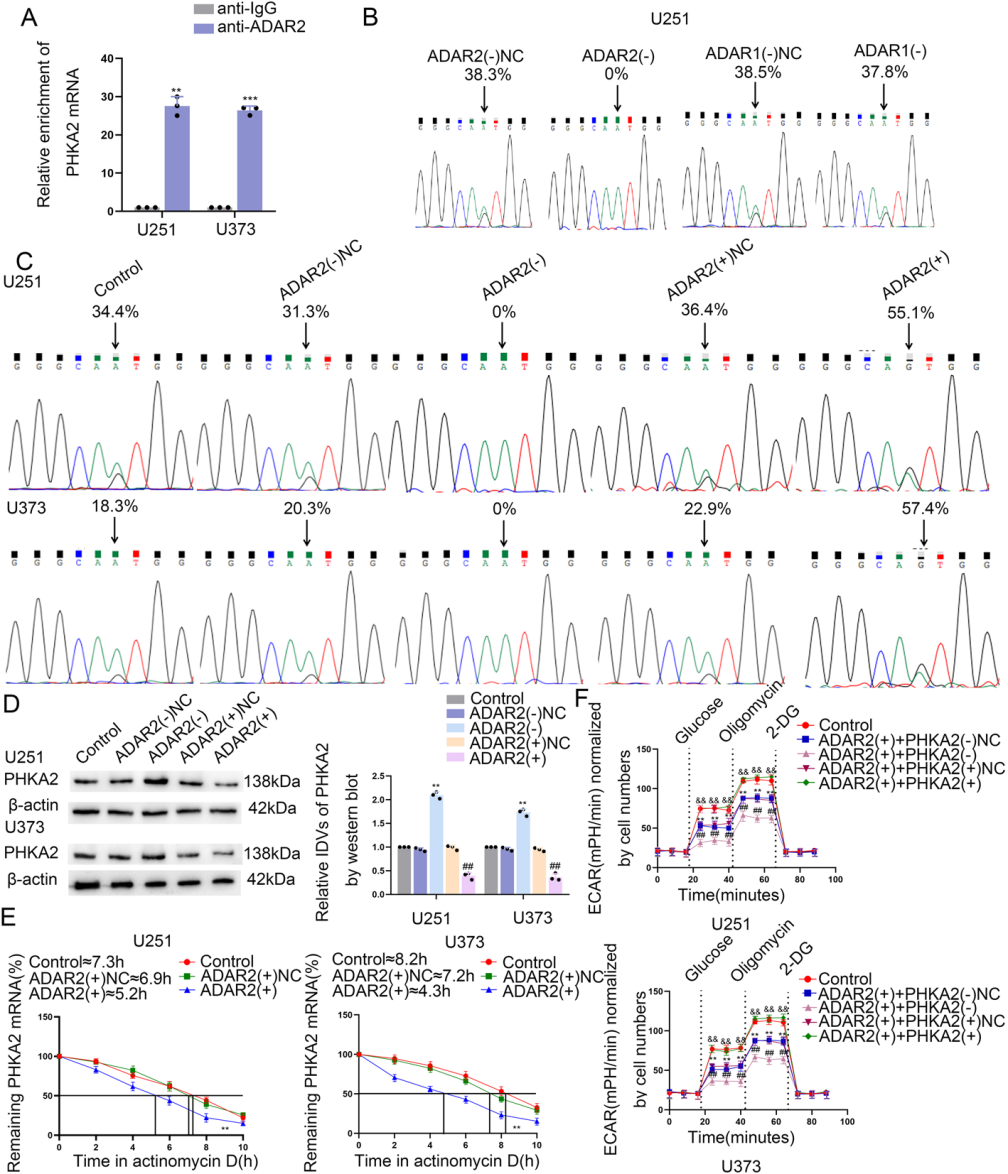
PHKA2 facilitated glycolipid metabolism of GBM cells by ADAR2-induced A-to-I RNA editing. **A** An enrichment of PHKA2 mRNA in ADAR2 immunoprecipitated samples via RNA immunoprecipitation (RIP) assay. Data presented as mean ± SD (*n* = 3, each group), ^**^P < 0.01 versus the anti-IgG group. **B** Sequence chromatograms of PHKA2 transcripts in U251 transduced with ADAR2(–)NC, ADAR2(–), or ADAR1(-)NC, ADAR1(–). Arrowheads indicate edited positions. Percentages indicate the calculated frequency of editing at selected positions. **C** Sequence chromatograms of the PHKA2 mRNA. Arrowheads indicate edited positions. **D** Western blot of ADAR2 and PHKA2 in U251 and U373 cells. Data presented as mean ± SD (*n* = 3, each group), ^**^P < 0.01 versus ADAR2(–)NC group;^##^*P* < 0.01 versus ADAR2( +)NC group. **E** The half-life of PHKA2 mRNA at different times treated by ActD with ADAR2 overexpression in U251 and U373 cells. Data presented as mean ± SD (*n* = 3, each group), ^**^P < 0.01 versus the ADAR2( +) group. **F** ECAR was used to measure the glycolysis and glycolytic capacity of U251 and U373 cells. Data presented as mean ± SD (*n* = 3, each group), ^**^P < 0.01 versus control group; ^##^*P* < 0.01 versus ADAR2( +) + PHKA2(-)NC group; ^&&^*P* < 0.01 versus ADAR2( +) + PHKA2( +)NC group. Statistical analysis was measured using the one-way ANOVA method.

The mechanism by which ADAR2 regulates PHKA2 expression was also investigated. RIP assays showed that PHKA2 mRNA can bind to ADAR2 (Fig. 5A). Editing sites for PHKA2 were identified in the RNA editing databases DARNED and REDIportal, suggesting PHKA2 as a potential A-to-I editing target gene. Given that A-to-I RNA editing events can potentially be facilitated by either ADAR2 or ADAR1, it is imperative that we eliminate the potential influence of ADAR1 on A-to-I RNA editing processes. The GBM-specific A-to-I RNA editing events that occurred in the 3′-UTR of PHKA2 mRNA were identified using Sanger sequencing. In the current study, no RNA editing changes were detected in U251 cells treated with ADAR2(−) NC, ADAR1(−), or ADAR1(−) NC, while a notable reduction in RNA editing was observed in U251 cells upon suppressing ADAR2 expression (Fig. 5B). The findings suggest that ADAR2 serves as the pivotal enzyme responsible for the specific A-to-I editing in the 3′-UTR region of PHKA2 mRNA, thereby playing a crucial role in GBM. Sanger sequencing analysis demonstrated that, upon knockdown of ADAR2, the A-to-I editing process within the 3′-UTR region of PHKA2 mRNA disappeared. Conversely, upon overexpression of ADAR2, the rate of A-to-I editing within the 3′-UTR region of PHKA2 mRNA was observed to be increased (Fig. 5C). The western blot assays demonstrated that the protein expression level of PHKA2 exhibited an elevation in response to the knockdown of ADAR2 expression, whereas it exhibited a decline upon the overexpression of ADAR2 (Fig. 5D). Given the possibility of A-to-I editing being implicated in the degradation of mRNA within the 3′-UTR region, a thorough examination of the half-life of PHKA2 mRNA was conducted. The results of the study demonstrate that overexpression of ADAR2 significantly reduced the half-life of PHKA2 mRNA in both U251 and U373 cells, thereby suggesting a critical regulatory role in this biological process (Fig. 5E). The above results demonstrate that ADAR2 could combine with PHKA2 and reduce PHKA2 mRNA stability through A-to-I editing, thereby reducing PHKA2 protein expression.

To further investigate the functional significance of PHKA2, we knocked down or overexpressed PHKA2 in ADAR2-overexpressed U251 and U373 cells. We observed that PHKA2 knockdown enhanced the inhibition of ADAR2 overexpressed on PKM2 and ACLY protein expression (Supplementary Fig. S5A), while aerobic glycolysis, lactate production, glucose utilization, lipogenesis, and cell proliferation were reduced (Fig. 5F, Supplementary Fig. S5B–G). However, it was observed that the overexpression of PHKA2 reversed the inhibitory impact of ADAR2 overexpression on the expression of PKM2 and ACLY, and glycolipid metabolism and GBM cell proliferation were significantly augmented (Fig. 5F, Supplementary Fig. S5A–G).

### EBF1 downregulation in GBM tissues and cells and EBF1 overexpression could suppress glycolipid metabolism and GBM cell proliferation

Transcription factors associated with PKM2 and ACLY were analyzed using Cistrome (https://www.dbtoolkit.cistrome.org). PKM2, which is encoded by PKM genes, was identified as being linked to a total of 101 genes. Additionally, ACLY was discovered to be associated with 66 genes. Notably, 19 genes exhibited potential associations with both PKM2 and ACLY (Supplementary Fig. S6A; Supplementary File 1). The expression of these 19 genes after PHKA2 knockdown at the transcriptional and translational levels is shown in Supplementary Fig. S6B, C. EBF1 was significantly upregulated upon PHKA2 knockdown; therefore, EBF1 was selected for further investigation.

**Fig. 6:**
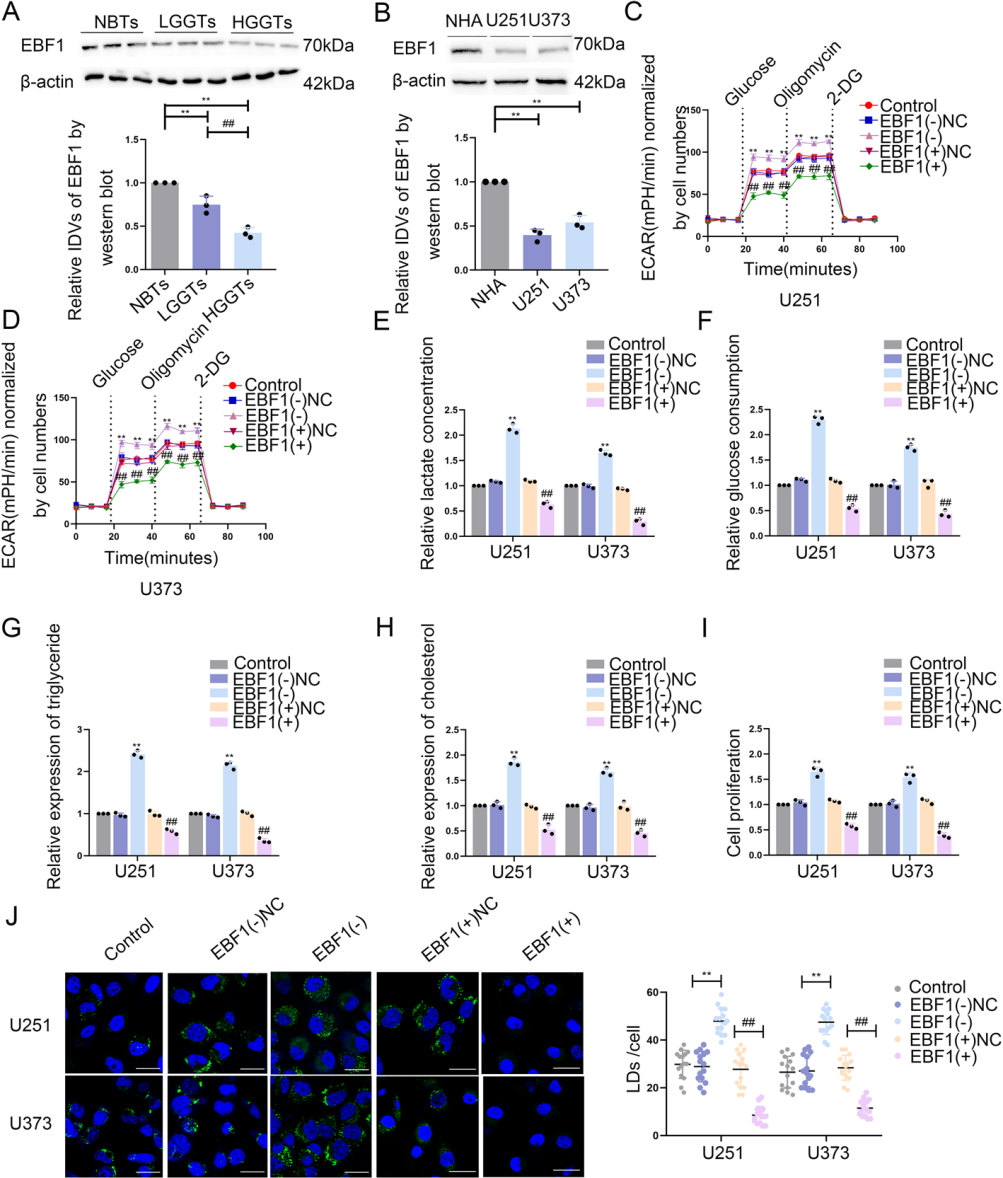
The expression of EBF1 and its effects on glycolipid metabolism in GBM. **A** EBF1 protein levels were analyzed in NBTs, LGGTs, and HGGTs by western blot. Data presented as mean ± SD (*n* = 3, each group), ^**^P < 0.01 versus the NBTs group; ^##^*P* < 0.01 versus the LGGs group. **B** EBF1 protein levels in NHA, U251, and U373 cells were detected via western blot. Data presented as mean ± SD (*n* = 3, each group), ^**^P < 0.01 versus the NHA group. **C**, **D** The extracellular acidification rate (ECAR) was measured to demonstrate the effects of EBF1 on glycolysis in U251 and U373 cells, and the glycolysis was calculated. Data presented as mean ± SD (*n* = 3, each group), ^**^P < 0.01 versus EBF1(–)NC group; ^##^*P* < 0.01 versus EBF1( +)NC group. **E**, **F** Lactate production and glucose uptake were measured in U251 and U373 cells after EBF1 knockdown or overexpression. Data presented as mean ± SD (*n* = 3, each group), ^**^P < 0.01 versus EBF1(–)NC group; ^##^*P* < 0.01 versus EBF1( +)NC group. **G**, **H** Intracellular triglyceride and cholesterol expression levels were measured to evaluate the effect of EBF1 on lipogenesis. Data presented as mean ± SD (*n* = 3, each group), ^**^P < 0.01 versus PHKA2(–)NC group; ^##^*P* < 0.01 versus PHKA2( +)NC group. **I** The effect of EBF1 on proliferation was analyzed via CCK-8 assay. ^**^P < 0.01 versus EBF1(–)NC group; ^##^*P* < 0.01 versus EBF1( +)NC group. Data presented as the mean ± SD (*n* = 3, each group). **J** Representative confocal fluorescence imaging of lipid droplets (LDs) stained by BODIPY 493/503 (green) in U251 and U373 cells. The nucleus (blue) was stained by DAPI. Scale bars = 10 µm. Data presented as mean ± SD (*n* = 15, each group). ^**^P < 0.01 versus EBF1(–)NC group; ^##^*P* < 0.01 versus EBF1( +)NC group. Statistical analysis was performed using the one-way ANOVA method

IF staining revealed that EBF1 is predominantly localized in the nuclei of GBM cells (Supplementary Fig. S6D). Compared with NBTs, the protein level of EBF1 in glioma tissues was observed to be relatively lower, with a further decline evident at higher pathological grades (Fig. 6A). In line with the glioma tissues, the GBM cells, such as U251 and U373, also exhibited significantly reduced EBF1 expression compared with NHAs (Fig. 6B). Subsequently, we conducted an analysis of the function of EBF1 in regulating glycolipid metabolism within GBM cells. Stable U251 and U373 cell lines with EBF1 knockdown or overexpression were developed. Supplementary Fig. S6E, F displays the EBF1 expression of transfected cells at transcriptional and translational levels. We observed significantly suppressed PKM2 and ACLY expression at the translational level (Supplementary Fig. S6G) and dramatically impaired glycolipid metabolism and markedly inhibited U251 and U373 cell growth (Fig. 6C–J). Conversely, increased PKM2 and ACLY expression, glycolipid metabolism, and GBM cell growth were observed after EBF1 knockdown (Supplementary Fig. S6G; Fig. 6C–J).

### PHKA2 phosphorylated EBF1 on Y256 and reduced EBF1 stability

**Fig. 7:**
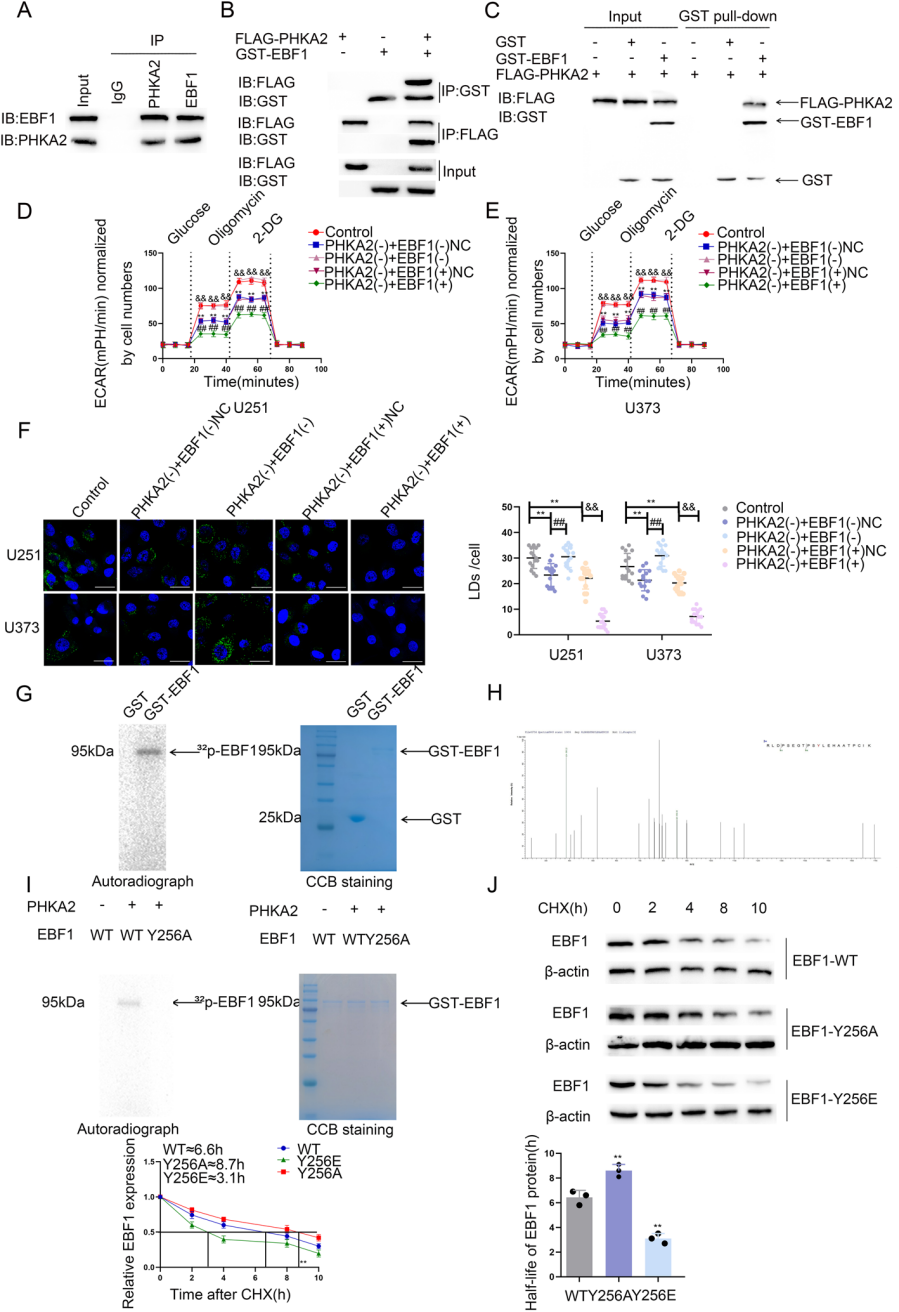
The effects of EBF1 phosphorylated by PHKA2 on glycolipid metabolism and proliferation in GBM. **A** Lysates of U251 cells were subjected to immunoprecipitation (IP) and immunoblotting (IB) with PHKA2 and EBF1 antibodies. **B** Lysates of 293 T cells transfected with FLAG-PHKA2 and GST-EBF1 plasmids were subjected to IP and IB with FLAG tag and GST tag antibodies. **C** The direct interaction between PHKA2 and EBF1 was confirmed by GST pull-down assays. GST protein functioned as a negative control. **D**, **E** ECAR was used to measure the glycolysis and glycolytic capacity of U251 and U373 cells. Data presented as mean ± SD (*n* = 3, each group), ^**^P < 0.01 versus control group; ^##^*P* < 0.01 versus PHKA2(–) + ENF1( +)NC group; ^&&^*P* < 0.01 versus PHKA2(–) + EBF1(–)NC group. **F** Representative confocal fluorescence imaging of LDs stained by BODIPY 493/503 (green) in U251 and U373 cells. The nucleus (blue) was stained by DAPI. Scale bars = 10 µm. Data presented as mean ± SD (*n* = 15, each group). ^**^P < 0.01 versus control group; ^##^*P* < 0.01 versus PHKA2(–) + EBF1( −)NC group; ^&&^*P* < 0.01 versus PHKA2(-) + EBF1( +)NC group. **G** Left panel, in vitro kinase assays were performed and detected by autoradiography (arrow, phosphorylated band). Right panel, proteins were visualized by Coomassie brilliant blue (CBB) staining. **H** The phosphorylated bands were subjected to mass spectrometry, and Y256 was identified. **I** In vitro kinase assays and CBB staining were conducted after Y256 mutation. **J** The effects of Y256 phosphorylation on EBF1 stability were detected by cycloheximide (CHX) chase assays. Data presented as mean ± SD (*n* = 3, each group). ^**^P < 0.01 versus EBF1-WT group. Statistical analysis was performed using the one-way ANOVA method

The possible interaction of PHKA2 with EBF1 was predicted using PPA-Pred2 (https://www.iitm.ac.in/bioinfo/PPA_Pred/) (Supplementary Fig. S7A). In U251 and U373 cells, IF staining revealed PHKA2 and EBF1 cytoplasm colocalization (Supplementary Fig. S7B). Additionally, co-IP assays showed an endogenous interaction of PHKA2 with EBF1 and FLAG-PHKA2 with GST-EBF1 in U251 and HEK-293 T cells, respectively (Fig. 7A, B). The PHKA2 and EBF1 direct binding was confirmed by an in vitro GST pull-down experiment using purified FLAG-PHKA2 and GST-EBF1 (Fig. 7C). Next, we knocked down or overexpressed EBF1 in PHKA2-deficient U251 and U373 cells. We found that EBF1 overexpression could enhance PKM2 and ACLY inhibition, glycolipid metabolism, and GBM cell proliferation in the PHKA2-knockdown group (Fig. 7D–F; Supplementary Fig. S7C–H), while EBF1 knockdown restored PKM2 and ACLY expression, increasing glycolipid metabolism and GBM cell proliferation (Fig. 7D–F; Supplementary Fig. S7C–H). Although EBF1 mRNA levels remained unchanged, the EBF1 protein was overexpressed following PHKA2 knockdown (Supplementary Fig. S7I, J). This observation indicates that the suppressive action of PHKA2 on EBF1 protein levels transpires following the translational process. A search using the phosphosite database (phosphosite.org/homeAction) identified multiple potential phosphorylation sites in EBF1 (Supplementary Fig. S7K). Together, these results indicate possible EBF1 phosphorylation by PHKA2. In vitro protein kinase assays confirmed that PHKA2 phosphorylates EBF1 (Fig. 7G). Using mass spectrometry analysis of the phosphorylated bands, the tyrosine at EBF1 position 256 (Y256) was identified as a specific PHKA2 phosphorylation site (Fig. 7H). Y256 mutation to alanine (Y256A) abolished the phosphorylated band (Fig. 7I). Subsequent in vivo experiments using customized specific phosphoantibodies showed that EBF1-Y256A phosphorylation was lower than that of the wild-type EBF1 (EBF1-WT), while EBF1-Y256E, a mutant in which Y256 is replaced by glutamate, had higher phosphorylation levels (Supplementary Fig. S7L). PHKA2 overexpression resulted in increased EBF1 phosphorylation at Y256, and this higher EBF1 phosphorylation level led to lower protein expression (Supplementary Fig. S7M, N). Furthermore, compared with EBF1-WT, EBF1-Y256A and EBF1-Y256E exhibited a significantly prolonged and shortened half-life, respectively (Fig. 7J).

### EBF1 inhibited PKM2 and ACLY transcription through directly binding to their promoters

**Fig. 8:**
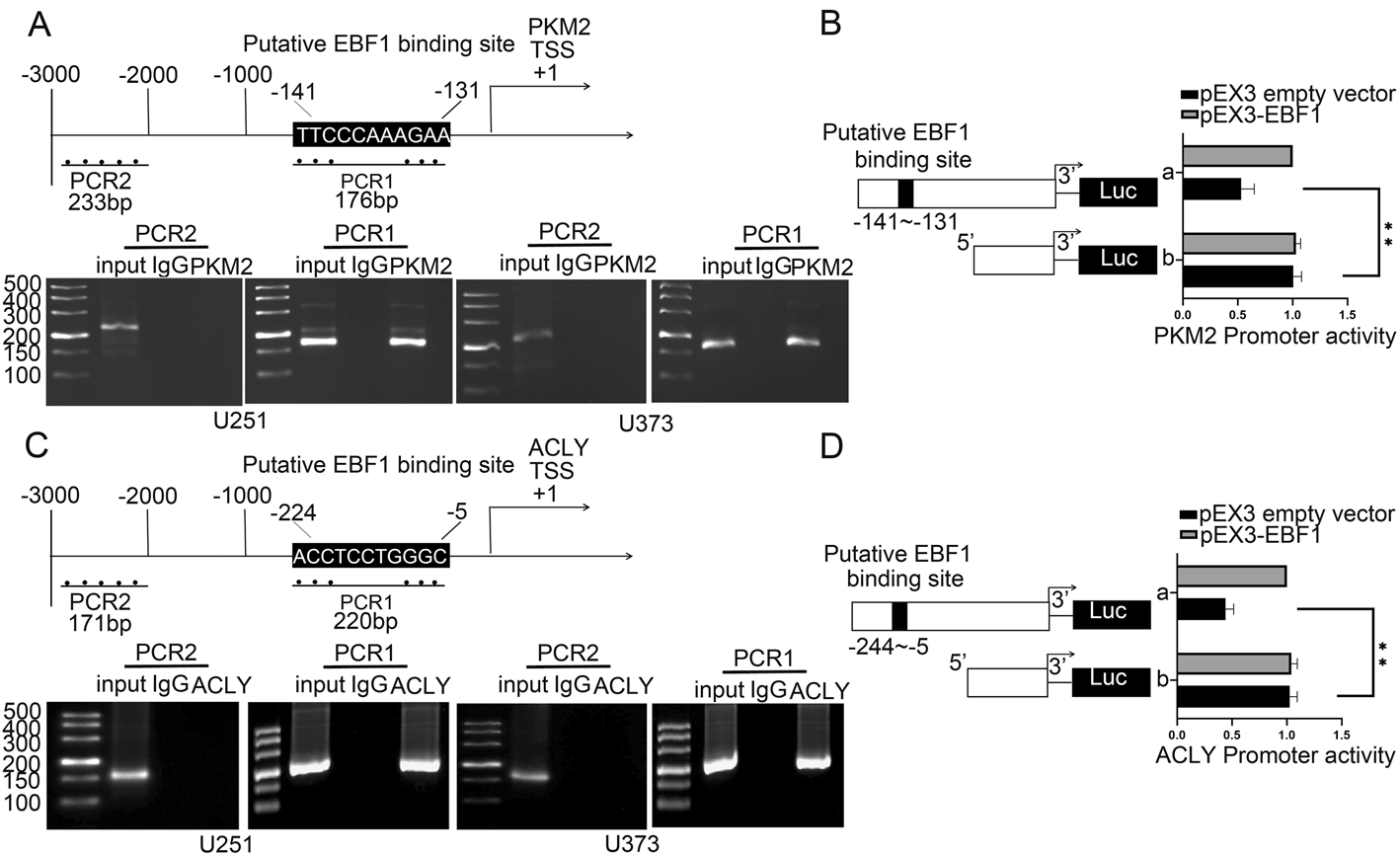
EBF1 directly bound to the promoter regions of PKM2, ACLY and transcriptionally suppressed their expression. **A** The putative EBF1 binding site is indicated in the PKM2 promoter region (above). Chromatin immunoprecipitation (ChIP) assay showed the products amplified putative EBF1-binding sites of PKM2 (below). **B** Schematic diagram of luciferase reporter construction and PKM2 relative luciferase activity measured in cells cotransfected with the PKM2 promoter (−1000 to 0 bp) (or PKM2 promoter-deleted putative EBF1 binding site) and pEX3 empty vector or pEX3-EBF1. Data presented as mean ± SD (*n* = 3, each group), ^**^P < 0.01 versus pEX3 empty vector group. **C** The putative EBF1 binding site is indicated in the ACLY promoter region (above). ChIP assay showed the products amplified putative EBF1-binding sites of ACLY (below). **D** Schematic diagram of luciferase reporter construction and ACLY relative luciferase activity measured in cells cotransfected with the ACLY promoter (−1000 to 0 bp) (or ACLY promoter-deleted putative EBF1 binding site) and pEX3 empty vector or pEX3-EBF1. Data presented as mean ± SD (*n* = 3, each group), ^**^P < 0.01 versus pEX3 empty vector group. One-way ANOVA was used for statistical analysis

The underlying molecular mechanisms behind PKM2 and ACLY suppression by EBF1 were further examined. Using the UCSC Genome Browser (http://genome.ucsc.edu/), we analyzed the transcription start sites of ACLY and PKM2, and several potential binding sites within the first 1000 bp of the ACLY and PKM2 promoter regions for EBF1 were discovered. Meanwhile, direct EBF1 binding to the PKM2 and ACLY promoter region was also observed in vitro (Fig. 8A–D). Further experiments revealed that EBF1 inhibited PKM2 and ACLY transcriptional expression. These findings demonstrate that the transcriptional regulation of PKM2 and ACLY is mediated by EBF1, which serves as a critical regulator in governing the biological behavior exhibited by GBM cells.

### Simultaneous SNORD113-3 and ADAR2 overexpression and PHKA2 knockdown suppressed tumor growth and prolonged nude mouse survival

**Fig. 9:**
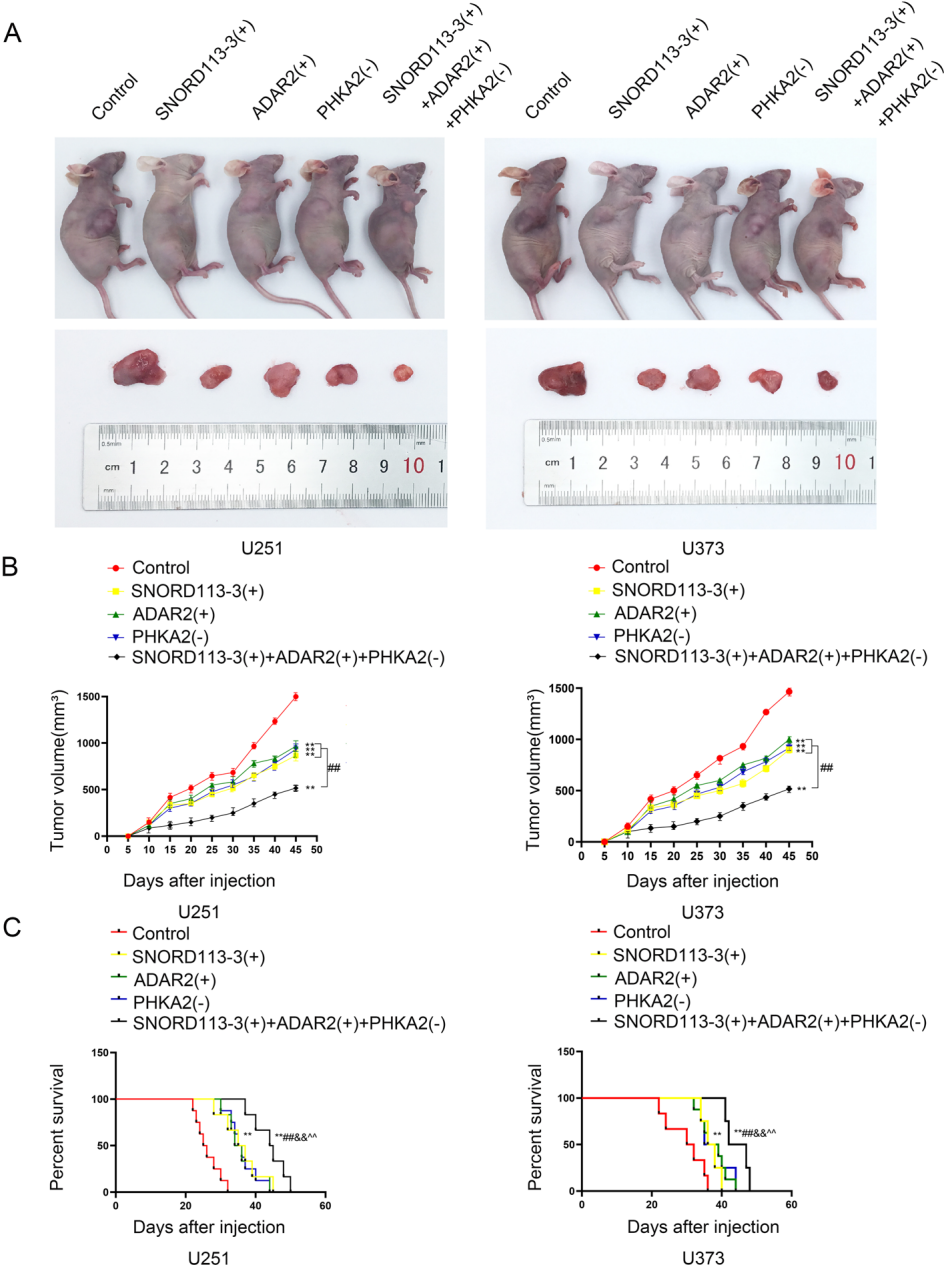
Overexpression of SNORD113-3 and ADAR2 with knockdown of PHKA2 suppressed tumor growth and prolonged survival in nude mice. **A** Subcutaneously xenografted nude mice injected with different treated cells are shown (above). Representative tumors from each group are shown (below). **B** Tumor growth curves are shown. Tumor size was recorded every 5 days, and tumors were extracted at 45 days after injection. ^**^P < 0.01 versus control group; ^##^*P* < 0.01 versus SNORD113-3(+) + ADAR2(+) + PHKA2(−) group by two-way ANOVA. **C** Survival curves of nude mice with orthotopic xenografts are shown. Data presented as mean ± SD (*n* = 8, each group). ^**^P < 0.01 versus control group; ^##^*P* < 0.01, ^&&^*P* < 0.01, ^^^^*P* < 0.01, SNORD113-3(+), ADAR2(+) or PHKA2(–) group compared with the SNORD113-3(+) + ADAR2(+) + PHKA2(–) group, respectively

To further examine the effect of SNORD113-3, ADAR2, and PHKA2 on tumor progression in vivo, GBM-SNORD113-3( +) cells, ADAR2( +) cells, PHAK2(–) cells, or a combination of them was injected to establish a mouse xenograft model. Compared with the control group, SNORD113-3( +), ADAR2( +), and PHKA2(–) significantly reduced the volume, with the smallest sizes observed in mice injected with PHKA2(–) or a combination of SNORD113-3( +) and ADAR2( +) (Fig. 9A, B).

**Fig. 10:**
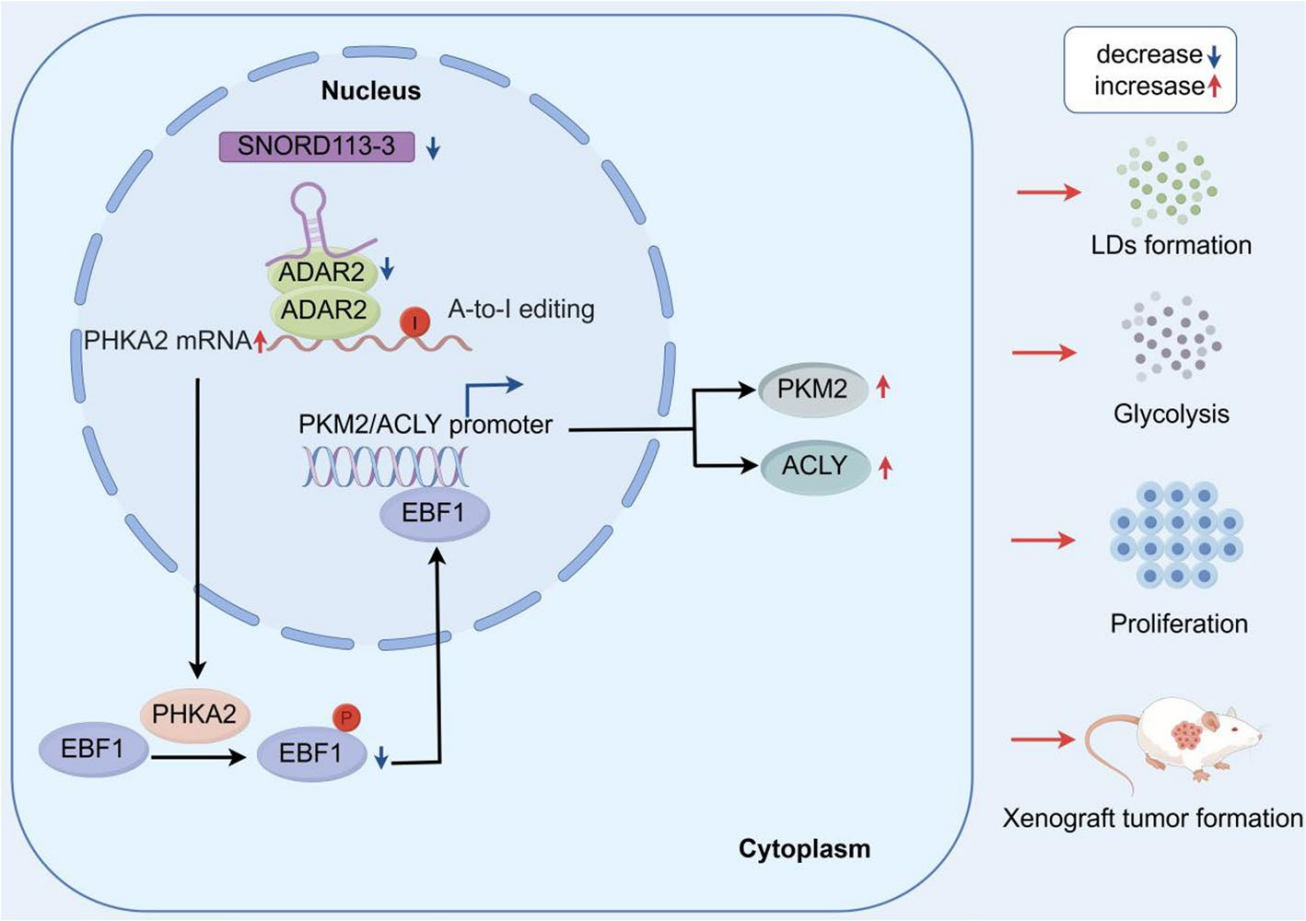
SNORD113-3 mediates ADAR2 A-to-I editing of PHKA2 mRNA, promoting EBF1-Y256 phosphorylation in the regulation of glycolipid metabolism and proliferation of GBM cells: schematic diagram

Next, we conducted a thorough analysis of the survival rates of mice implanted with the specified GBM cells. Compared with the control group, mice injected with SNORD113-3(+), ADAR2(+), and PHKA2(–) showed significantly longer survival times. Notably, the mice injected with all three factors displayed the longest survival duration. (Fig. 9C). Figure 10 shows a schematic diagram of the molecular mechanism by which SNORD113-3 mediates ADAR2 A-to-I RNA editing of PHKA2 mRNA to promote EBF1-Y256 phosphorylation, regulating glycolipid metabolism and GBM cell growth.

## Discussion

Recently, several studies have suggested the regulatory effect of snoRNAs on tumor cell energy metabolism and growth. For example, SNORD89 is upregulated in endometrial cancer cells, enhancing cancer cell proliferation and migration [24]. U3 SNORNA regulates GBM cell aerobic glycolysis and proliferation [25]. Therefore, to screen for snoRNAs that affect GBM glycolipid metabolism, we screened the 10 snoRNAs with differential expression in GBM using the TCGA database for predictions. After further comparison of these ten snoRNAs in lactate generation and glucose uptake, we selected SNORD113-3, with the strongest GBM glycolysis inhibition, for further study. In GBM tissues and cells, downregulation of SNORD113-3 has been observed. Additionally, overexpression of SNORD113-3 has been demonstrated to effectively suppress glycolipid metabolism and cellular proliferation in GBM cells. Thus, SNORD113-3 could potentially act as a tumor suppressor gene, playing a role in the regulation of glycolipid metabolism and cellular proliferation in GBM cells. However, the precise mechanism underlying the suppressive effect of SNORD113-3 on the expression of PKM2 and ACLY proteins, leading to the inhibition of glycolipid metabolism as well as proliferation in GBM cells, remains unclear. Therefore, further detailed explorations are necessary to elucidate this phenomenon.

In the present study, genomic microarray analysis was performed on GBM cells by overexpressing SNORD113-3, and the four genes with the most significant changes were identified. After further comparison of the roles of the four genes in lactate production and glucose uptake, ADAR2 expression had the most notable difference, whereas lactate production and glucose uptake were the most significant. Therefore, ADAR2 was selected as a potential target and used for further investigation.

Recent studies indicate that ADAR2 plays a role in the A-to-I editing process of GBM and potentially influences the malignant biological behavior exhibited by GBM cells. For example, A-to-I editing which was ADAR2-mediated was reduced significantly in GBM [26]. Furthermore, ADAR2 mediates A-to-I editing of CDC14B pre-mRNA to inhibit GBM cell proliferation [27]. We observed that ADAR2 has low expression in gliomas, and ADAR2 overexpression can significantly inhibit glycolipid metabolism as well as cell proliferation in GBM cells. Thus, it is conceivable that ADAR2 may serve as a tumor suppressor gene, regulating glycolipid metabolism and proliferation in GBM cells.

The key function of C/D box snoRNAs is to direct rRNA site 2′-O-methylation [28]. However, no 2′-O-methylation site was predicted at the ADAR2 binding site. Macromolecular complex assembly is fundamental for the proper functioning of many proteins [29, 30]. The enzyme activity of ADAR2 is contingent upon its ability to form homodimers [31, 32]. Furthermore, LNC-SNO49AB improved ADAR1 enzymatic activity by promoting ADAR1 homodimerization in leukemia cell lines [33]. Therefore, we explored whether SNORD113-3 promotes ADAR2 protein expression by promoting ADAR2 homodimer formation in GBM cells. We demonstrate that SNORD113-3 is capable of forming a functional structure in conjunction with the ADAR2 dimer. Furthermore, we establish that an elevation in SNORD113-3 expression can stimulate the formation of ADAR2 dimers. As the homodimer formation of the protein enhances protein stability [34], the present study aimed to further investigate whether SNORD113-3 enhances ADAR2 stability by promoting ADAR2 dimer formation, subsequently increasing ADAR2 protein expression. After overexpressing SNORD113-3, the half-life of ADAR2 was extended notably, suggesting an increase in ADAR2 stability. Consequently, SNORD113-3 overexpression has the potential to facilitate ADAR2 dimer formation and enhance ADAR2 stability, ultimately augmenting ADAR2 protein expression, indicating that snoRNAs may also influence the assembly of other proteins in GBM.

This study used a genome microarray platform analysis to explore the downregulation of genes in GBM cells caused by ADAR2. The analysis revealed that the most significant change occurred in PHKA2. The phosphorylase kinase PHKA2 has been implicated extensively in glycogen storage disease [35]. To the utmost of our comprehension, this report marks the inaugural instance of documenting the upregulation of PHKA2 in tissues and cells pertaining to GBM. Additionally, our findings demonstrate a significant positive association between PHKA2 expression levels and the pathological grade. Furthermore, our research indicates that PHKA2 functions to augment glycolipid metabolism and promote the proliferative capacity of GBM cells.

Cancer arises from alterations in genomic information [36]. Epitranscriptome changes can be induced by methylation modification and RNA editing [37, 38]. Although epitranscriptome alterations contribute to tumorigenesis [39], their specific regulatory mechanisms remain unclear. A-to-I editing is a key epitranscriptome phenomenon crucial for cancer development. Specifically, the circ-NEIL3 regulatory mechanism promotes the multiplication of pancreatic ductal adenocarcinoma cells by means of A-to-I RNA editing [40]. A-to-I RNA editing of the bladder cancer-associated protein BLCAP promotes bladder cancer cell proliferation [41]. ADAR1 mediates A-to-I RNA editing of GM2A to promote in vivo tumorigenesis [42]. Our findings indicate that ADAR2, rather than ADAR1, possesses the ability to initiate A-to-I RNA editing within the 3′-UTR region of PHKA2 mRNA. This editing process subsequently leads to modifications in PHKA2 protein levels, which may be attributed to the potential role of A-to-I editing in mRNA degradation within the 3′-UTR region [43]. In the current study, rigorous RNA half-life experiments unequivocally demonstrated a notable reduction in the half-life of PHKA2 mRNA upon overexpression of ADAR2. This observation indicates that ADAR2 impairs PHKA2 mRNA stability. Consequently, ADAR2 mediates the reduction of PHKA2 mRNA stability through A-to-I editing, leading to accelerated degradation and subsequent alterations in PHKA2 protein levels. This indicates that the integrated glycolipid metabolism regulatory mechanism of A-to-I editing could offer innovative insights that could facilitate development of targeted therapies for GBM.

EBF1 functions as a transcription factor, which, in conjunction with PAX5, significantly regulates the developmental process of normal and malignant B-cell acute lymphoblastic leukemia by binding to MYC gene regulatory elements [44]. EBF1 has also been shown to regulate telomerase catalytic subunit expression in gastric cancer [45] and mediate ribosome assembly factor PNO1 upregulation, which suppresses p53 signaling pathway regulation, promoting colorectal cancer cell proliferation [46]. Our observations reveal that the expression of EBF1 is downregulated in GBM tissues and cells, exhibiting a decreasing trend as the pathological grade escalates. Furthermore, the overexpression of EBF1 has been demonstrated to suppress glycolipid metabolism and GBM cell proliferation.

We observed that PHKA2 decreases EBF1 protein expression without affecting EBF1 mRNA levels. EBF1 knockdown reverses the suppressive effects of PHKA2 knockdown on glycolipid metabolism and GBM cell proliferation, indicating that PHKA2 inhibits such processes by downregulating EBF1 protein levels. PHKA2 domain analysis suggests that the mechanism by which PHKA2 reduces EBF1 protein levels may involve phosphorylation modifications. Previous research on the transcriptional repressor protein YY1 confirmed that phosphorylation can alter protein stability [47]. We have successfully demonstrated the phosphorylation of EBF1 Y256 both in vitro and in vivo, and further elucidated that an elevated phosphorylation level at Y256 leads to a reduction in EBF1 protein expression. This indicates that PHKA2 reduces EBF1 expression through phosphorylation at Y256, decreasing EBF1 stability.

Glycolipid metabolism reprogramming is a characteristic of altered energy metabolism in GBM cells [48]. Increased PKM2 expression could enhance aerobic glycolysis and result in macromolecule (citrate and acetyl-CoA) production in GBM cells [49]. The glycolysis-produced citrate can be transported from mitochondria to the cytoplasm by ACLY, and then ACLY could catalyze acetyl-CoA production using citrate [50]. Acetyl-CoA is a key raw material for in vivo lipid and cholesterol synthesis. Significantly enhanced and impaired PKM2 and ACLY expression was observed after EBF1 knockdown and overexpression, respectively. Furthermore, upon meticulous analysis, putative binding sites for EBF1 were detected within the promoter regions of PKM2 and ACLY. Through direct interaction with these promoter regions, EBF1 exhibits the capacity to downregulate the transcription of PKM2 and ACLY. Therefore, EBF1 regulates glycolipid metabolism and GBM cell proliferation by inhibiting PKM2 and ACLY transcription and expression.

Ultimately, the in vivo experiments demonstrated that overexpression of SNORD113-3 or ADAR2, as well as knockdown of PHKA2, effectively suppress the development of GBM in vivo and enhance the survival outcomes of mice harboring GBM. SNORD113-3 and ADAR2 overexpression combined with PHKA2 knockdown had stronger tumor suppressive effects. The results highlight the potential utility of SNORD113-3, ADAR2, and PHKA2 as therapeutic targets for GBM.

## Conclusions

This study elucidates the complex molecular regulatory network in GBM involving SNORD113-3, ADAR2, PHKA2, and EBF1. PHKA2 was identified as an oncogenic factor, whereas SNORD113-3, ADAR2, and EBF1 acted as tumor suppressors. PHKA2 knockdown, or SNORD113-3, ADAR2, and EBF1 overexpression, dramatically suppressed glycolipid metabolism and GBM cell proliferation. SNORD113-3 promotes ADAR2 protein expression by promoting ADAR2 homodimer formation. ADAR2 mediates the A-to-I RNA editing of PHKA2 mRNA. This upregulates PHKA2 protein expression and enhances glycolipid metabolism and GBM cell proliferation. In the GBM cytoplasm, PHKA2 phosphorylates EBF1 at Y256, decreasing EBF1 stability and expression. EBF1 transcriptionally represses PKM2 and ACLY expression. EBF1 downregulation in GBM cells led to an increase in PKM2 and ACLY expression, promoting glycolipid metabolism and GBM cell proliferation. An axis comprising SNORD113-3/ADAR2/PHKA2/EBF1 is formed, jointly regulating glycolipid metabolism and GBM cell proliferation. These conclusions practically explain the complex molecular mechanisms underlying GBM pathogenesis and offer potential therapeutic targets for GBM.

### The constitution of the Ethics Committee of Shengjing Hospital

In order to ensure the scientific nature and benefit of medical scientific research projects, optimize the health interests and interests of the participants, reduce or avoid research harm, according to the state food and drug administration, the national health committee issued the drug clinical trial quality management specification (2020), the former national development planning commission issued the biomedical research ethics review method (2016), the World Medical Association formulated the Declaration of Helsinki (2013) and the Basel Declaration (2010), and other principles and norms, formulate the medical ethics committee work as follows:

General: medical ethics committee to maintain the dignity, rights, safety and welfare of the participants, independent, fair and timely review of the proposed ethics, biomedical research activities, and the consent and consent and ongoing research activities for regular ethical evaluation. It has the responsibility to fully consider the interests of the participants in the research activities and their related communities, while considering the interests and needs of the researchers, and to comply with the relevant laws and regulations.

### Clinical specimens

We obtained normal brain and glioma specimens from China Medical University’s Affiliated Hospital for Neurosurgery. This was approved by the Ethics Committee of China Medical University’s affiliated Shengjing Hospital, and all patients or their families signed the informed consent form (approval no. 2023PS957K, date 14 July 2023).

### Mouse xenograft model

BALB/c nude mice (4 weeks old) were obtained from Vital River Laboratory in Beijing for in vivo studies. All procedures adhered to the protocol approved by the Ethics Committee of China Medical University (approval no. 2023PS960K, date 14 July 2023).

## Supplementary Information

Additional file 1.

Additional file 2.

Additional file 3.

Additional file 4.

Additional file 5.

Additional file 6.

Additional file 7.

Additional file 8.

Additional file 9.

## Data Availability

The data that support the findings of this study are available from the corresponding author upon reasonable request.

## Abbreviations

GBM: Glioblastoma multiforme
CNS: Central nervous system
NBTs: Normal brain tissues
NHA: Normal human astrocytes
PKM2: Pyruvate kinase M2
ACLY: ATP citrate lyase
rRNA: Ribosomal RNA
PHKA2: Phosphorylase kinase regulatory subunit alpha 2
EBF1: EBF transcription factor 1
FISH: Fluorescence in situ hybridization
IF: Immunofluorescence
ChIP: Chromatin immunoprecipitation
ECAR: Extracellular acidification rate
NC: Negative control
GST: Glutathione-*S*-transferase
SD: Standard deviation
EBF1-WT: Wild-type EBF1
