# Supplementary figures and images for: Effect of SNORD113-3/ADAR2 on glycolipid metabolism in glioblastoma via A-to-I editing of PHKA2
Source: Cell Mol Biol Lett. 2025 Jan 10;30:5. doi: 10.1186/s11658-024-00680-9 (PMC11724473; doi:10.1186/s11658-024-00680-9)

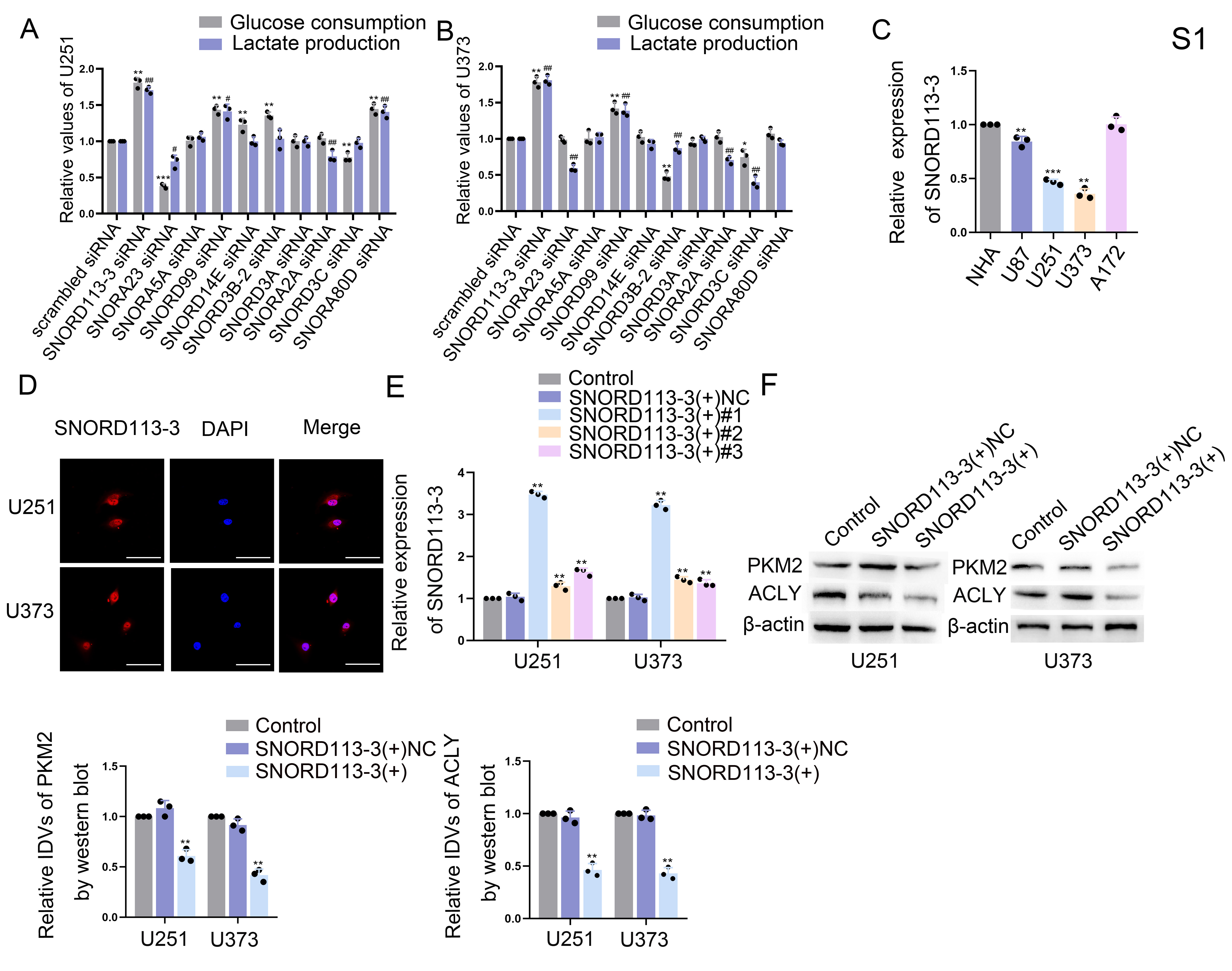

Supplement: Supplementary file 1 — Additional file 1. [file 11658_2024_680_MOESM1_ESM.jpg]

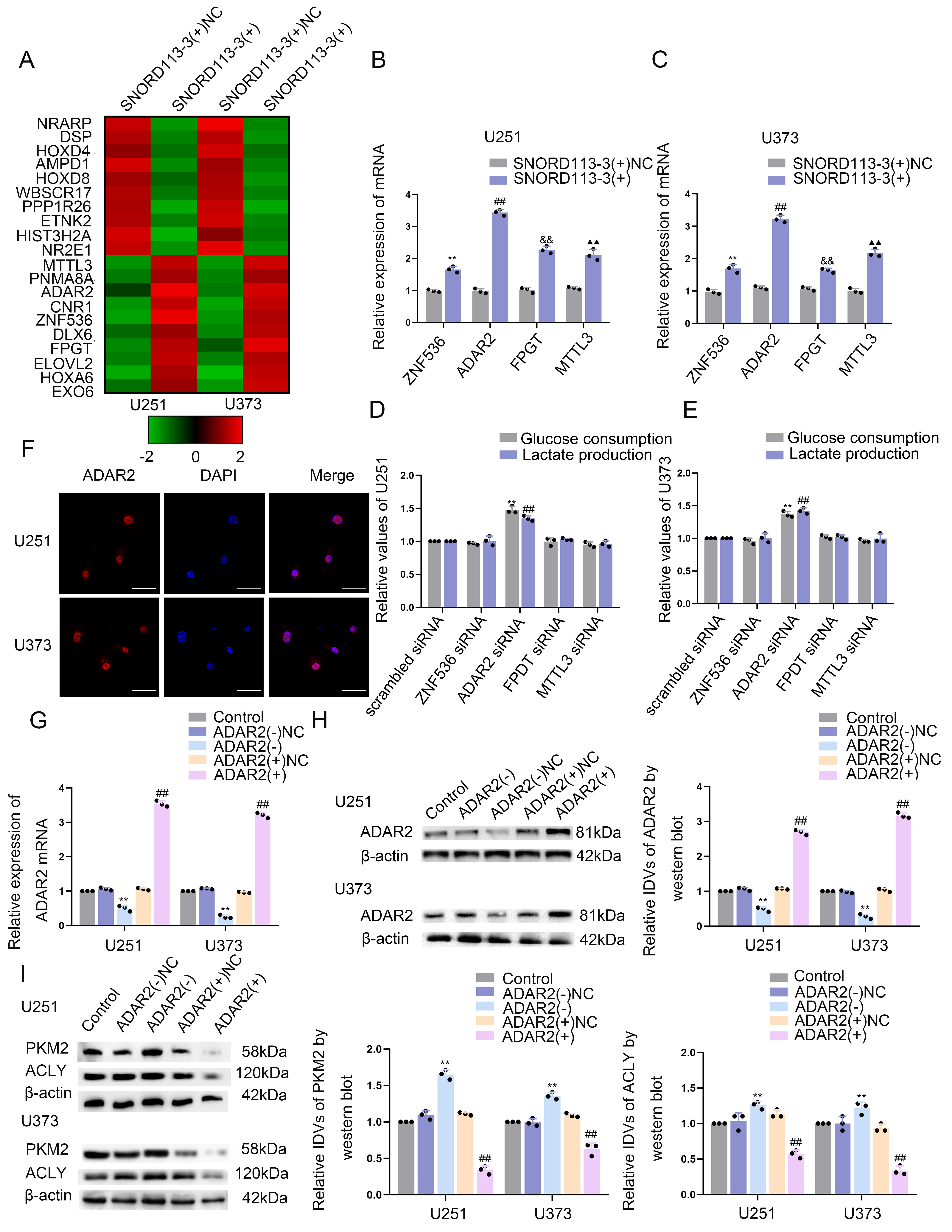

Supplement: Supplementary file 2 — Additional file 2. [file 11658_2024_680_MOESM2_ESM.jpg]

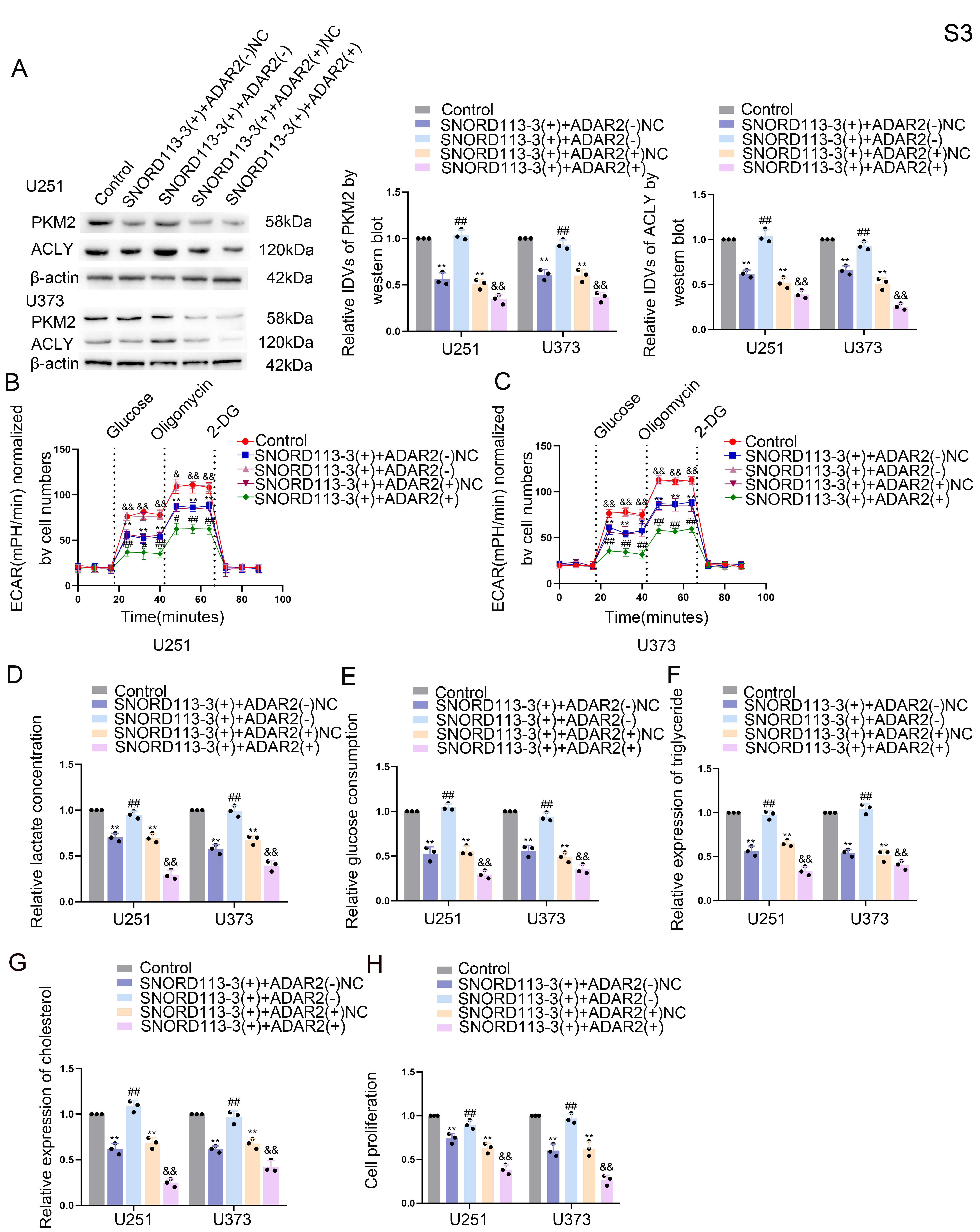

Supplement: Supplementary file 3 — Additional file 3. [file 11658_2024_680_MOESM3_ESM.jpg]

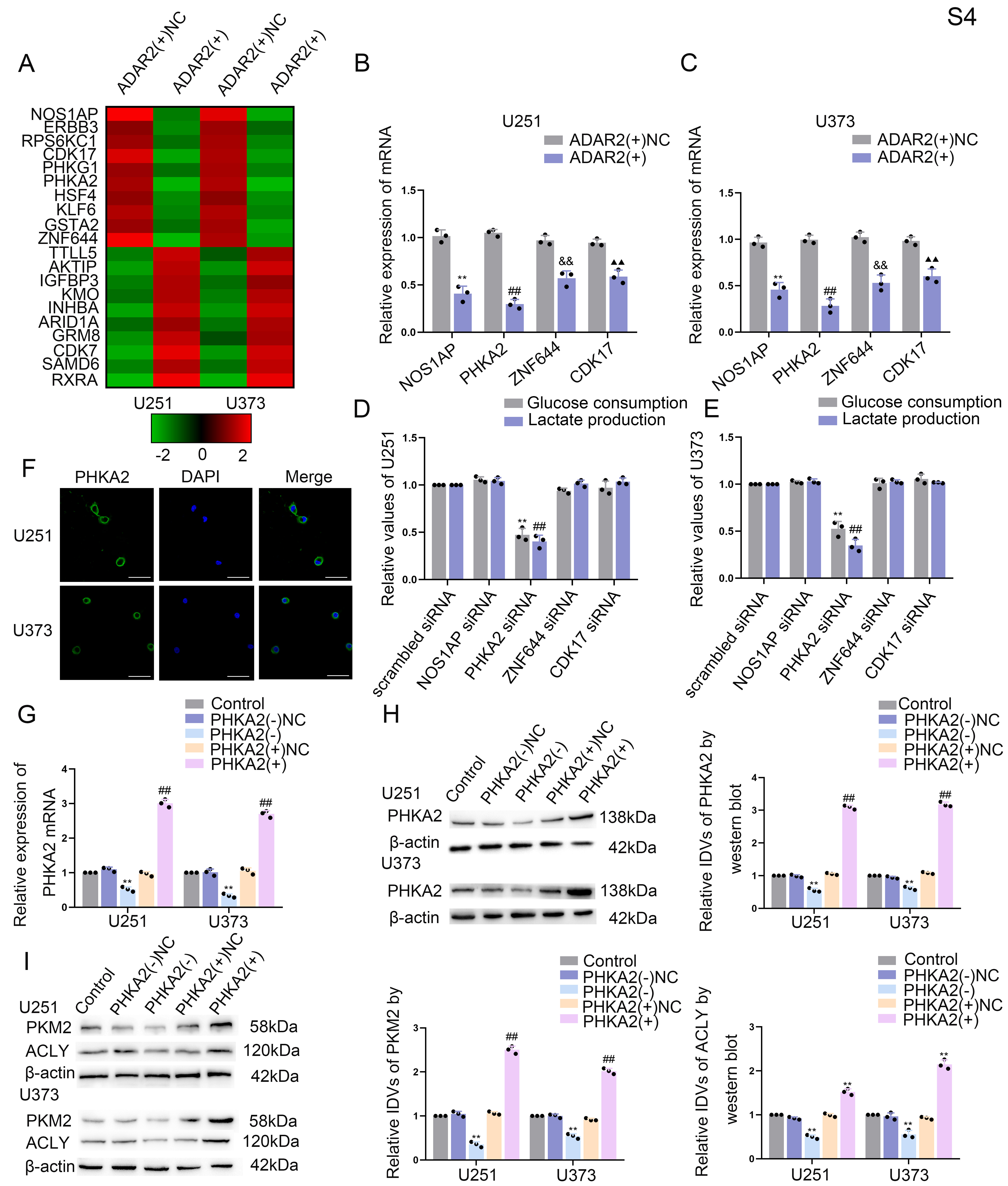

Supplement: Supplementary file 4 — Additional file 4. [file 11658_2024_680_MOESM4_ESM.jpg]

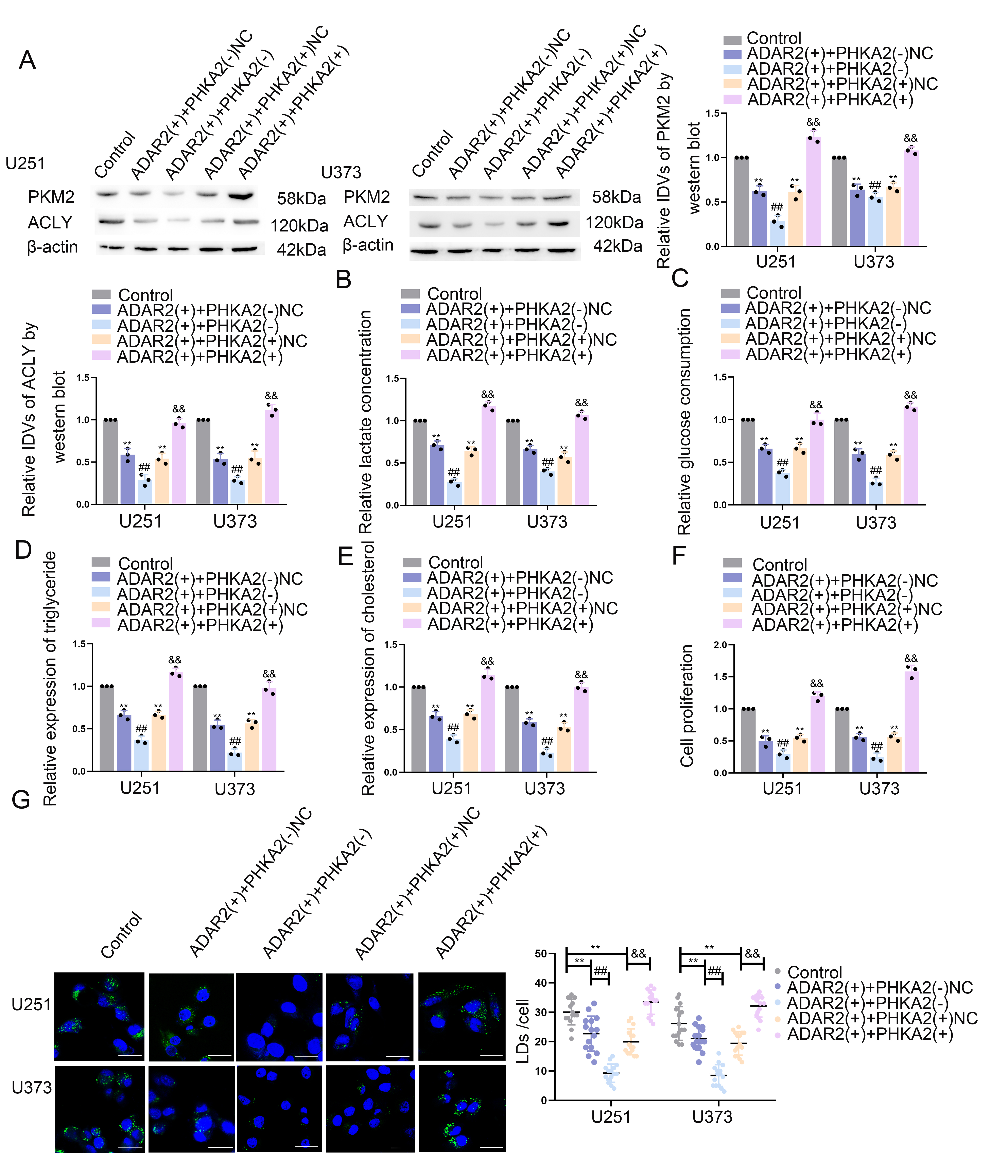

Supplement: Supplementary file 5 — Additional file 5. [file 11658_2024_680_MOESM5_ESM.jpg]

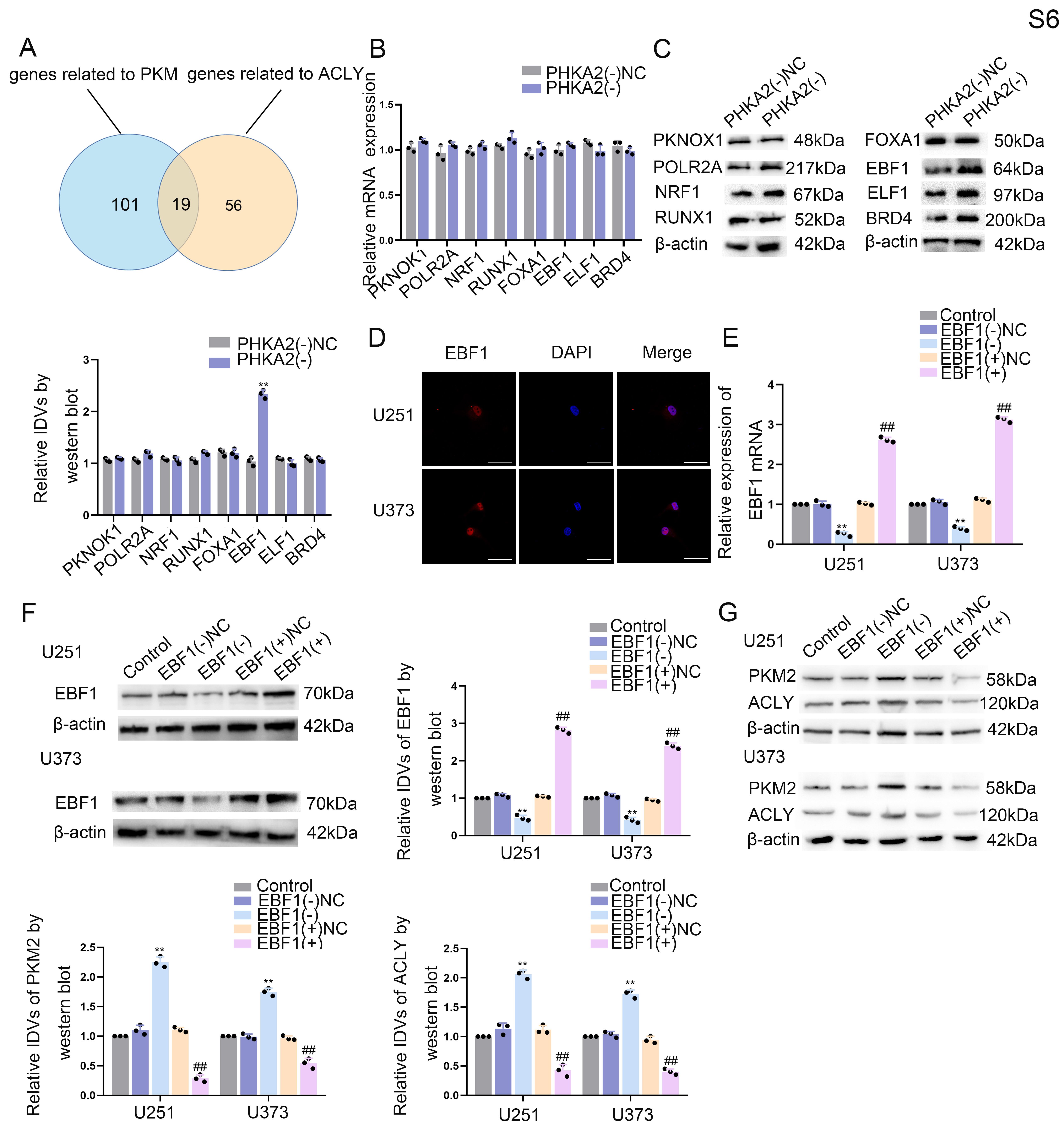

Supplement: Supplementary file 6 — Additional file 6. [file 11658_2024_680_MOESM6_ESM.jpg]

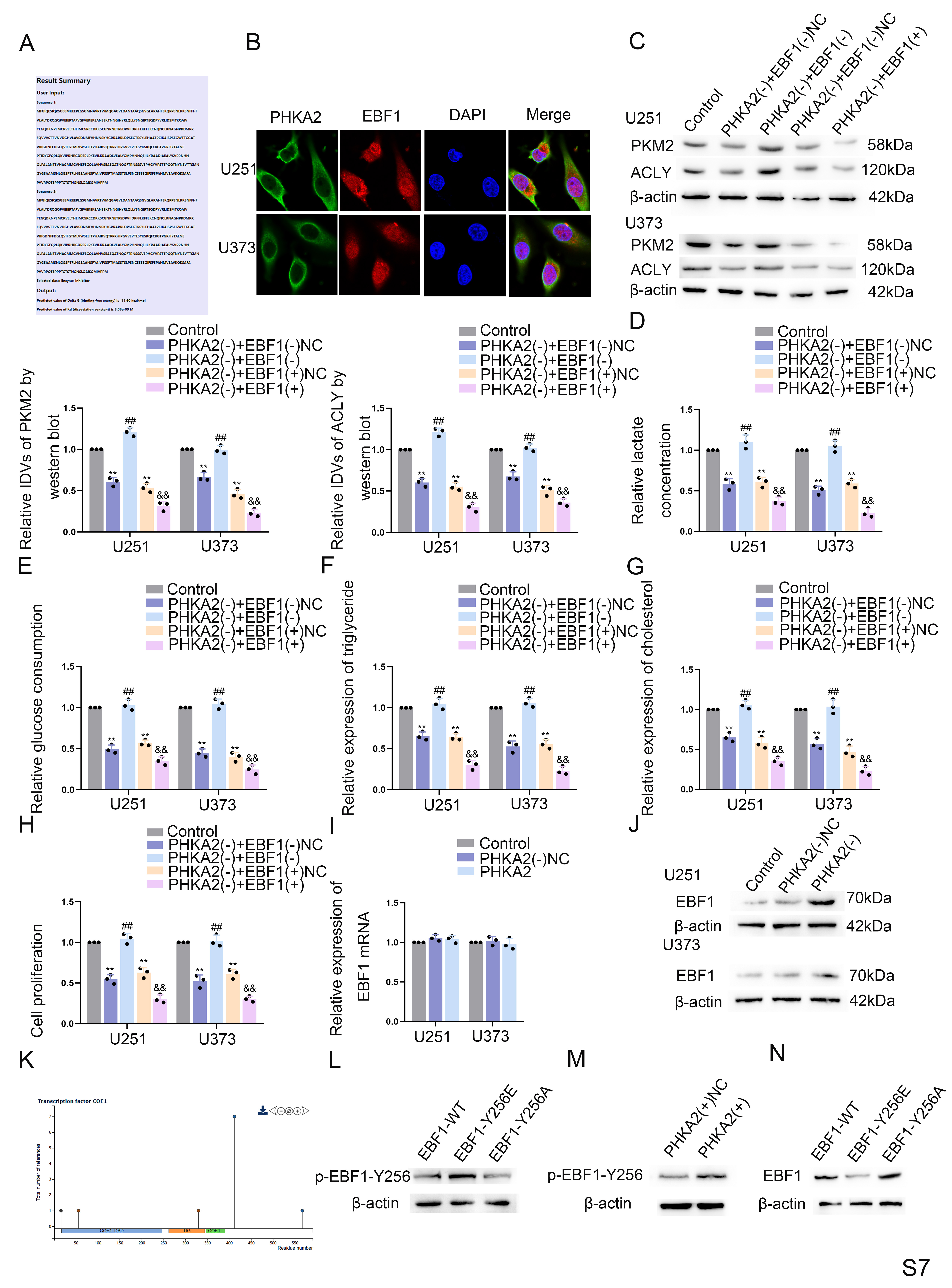

Supplement: Supplementary file 7 — Additional file 7. [file 11658_2024_680_MOESM7_ESM.jpg]
